# Age-related profiling of DNA methylation in CD8+ T cells reveals changes in immune response and transcriptional regulator genes

**DOI:** 10.1038/srep13107

**Published:** 2015-08-19

**Authors:** Liina Tserel, Raivo Kolde, Maia Limbach, Konstantin Tretyakov, Silva Kasela, Kai Kisand, Mario Saare, Jaak Vilo, Andres Metspalu, Lili Milani, Pärt Peterson

**Affiliations:** 1 Molecular Pathology, Institute of Biomedical and Translational Medicine, University of Tartu, Tartu, Estonia; 2 Institute of Computer Science, University of Tartu, Tartu, Estonia; 3 Estonian Genome Center, University of Tartu, Tartu, Estonia; 4 Institute of Molecular and Cell Biology, University of Tartu, Tartu, Estonia

## Abstract

Human ageing affects the immune system resulting in an overall decline in immunocompetence. Although all immune cells are affected during aging, the functional capacity of T cells is most influenced and is linked to decreased responsiveness to infections and impaired differentiation. We studied age-related changes in DNA methylation and gene expression in CD4+ and CD8+ T cells from younger and older individuals. We observed marked difference between T cell subsets, with increased number of methylation changes and higher methylome variation in CD8+ T cells with age. The majority of age-related hypermethylated sites were located at CpG islands of silent genes and enriched for repressive histone marks. Specifically, in CD8+ T cell subset we identified strong inverse correlation between methylation and expression levels in genes associated with T cell mediated immune response (*LGALS1, IFNG, CCL5, GZMH, CCR7, CD27* and *CD248*) and differentiation (*SATB1, TCF7, BCL11B* and *RUNX3*). Our results thus suggest the link between age-related epigenetic changes and impaired T cell function.

Ageing progressively affects the activity of the immune system. The age-related decline in immune responses, also termed immunosenescence, is prominent in the adaptive immune system, where T cells appear to be most affected during ageing^1^,^2^. Both CD4+ and CD8+ T cell compartments change with age, however, the CD8+ T cell population in particular becomes enriched for a high proportion of terminally differentiated effector cells, which have lower responsiveness to antigens^1^. Chronic infection with cytomegalovirus (CMV) is implicated in the age-dependent expansion of the CD8+ T cell subset^3^,^4^, and in the older adults, 25–50% of the CD8+ cells can be specific to CMV^5^,^6^,^7^. A typical feature of ageing is a chronic, low-grade inflammatory status, named inflamm-ageing, which is characterized by a general increase in proinflammatory cytokines^8^,^9^. The increased expression of the proinflammatory markers suggests that immunosenescence is driven by a chronic antigenic load, which induces the proinflammatory phenotype^10^. Accordingly, the concept of an immune risk profile, associated with higher mortality rates, has been proposed. This is composed of depleted naïve T cell numbers, an inverted CD8+/CD4+ T cell ratio, low responses to mitogen stimulation, increased population of anergic terminally differentiated CD8+ T cells and increased pro-inflammatory cytokine expression^11^.

Age-related transcriptome marker analyses in peripheral blood leukocytes (PBL) or mononuclear cells have revealed changes in gene expression levels and aberrant mRNA splicing^12^,^13^,^14^, with gender-specific effects^15^ and dependence upon CMV phenotype^16^. Several studies have used peripheral blood cells for the analysis of DNA methylation changes during ageing, and have revealed differentially methylated CpG sites^17^,^18^, hypermethylation of CpG islands in promoters^19^,^20^, existence of mega-base scale hypomethylated blocks^21^, and proposed methylation biomarkers as predictors of age^22^,^23^,^24^. However, it should be noted that the transcriptional and epigenetic analyses in human peripheral blood samples are affected by the numerical alteration of specific cell subsets in blood including T cells, B cells, monocytes and granulocytes^25^,^26^. Even if normalization tools are applied^27^, the use of purified cell subsets is superior to the identification of methylome changes and their correlation to cell-type specific functions.

In light of these considerations we focused on sorted CD4+ and CD8+ T cells and aimed to identify whether the DNA methylation changes are involved in development of age-related decrease of T cell responsiveness. We purified DNA from PBL, CD4+ and CD8+ T cells from younger and older individuals, and compared their genome-wide DNA methylation differences. We correlated the age-related methylation changes with gene expression levels and histone modifications in the same regions. Our genome-wide analyses indicate for the first time in CD8+ T cell subset a strong age-related correlation of DNA methylation and the expression of genes having functional role in controlling T cell immune responses and their differentiation.

## Results

### Age-related methylation changes in PBL, CD4+ and CD8+ T cells

**Figure 1:**
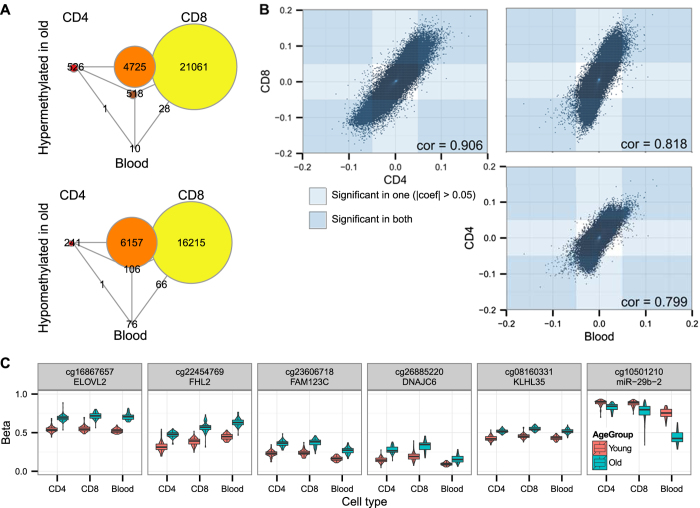
Differentially methylated sites in PBL, CD4+ and CD8+ T cells. (**A**) Venn diagram showing the overlaps between age-related differentially methylated sites in PBL, CD4+ and CD8+ T cells. The circles in the corners show the number of CpG sites unique to each cell type and the intermediate circles show the number of overlapping sites between the respective cell types. (**B**) Correlation of fold changes for all sites. The regions of the plot that correspond to |*fold change*| > 0.05 are indicated by blue rectangles (t-test adjusted p-value < 0.01). (**C**) Methylation levels of the top differentially methylated CpG sites in CD4+ and CD8+ T cells and in PBL samples.

We studied the methylomes in PBL, and CD4+ and CD8+ T cells from 50 younger (age 22–34) and 50 older (age 73–84) individuals using the Illumina HumanMethylation450 arrays. For the differential methylation analysis, we used a linear model to remove confounding effects (see Materials and Methods for details). As the numbers of leukocyte subsets in blood vary greatly with age and skew the analysis of PBL samples, we applied additional normalization based on cell numbers in PBL samples to remove the effect of variable numbers of granulocyte, lymphocyte and monocyte subsets in each PBL sample based on measured and estimated hematological cell counts. After the normalization for variation in cell counts, we confirmed only a small proportion of the differentially methylated sites in PBL (7725 before and 806 after the normalization). In contrast, we identified 12,275 and 48,876 differentially methylated CpG sites in the purified CD4+ and CD8+ T cells (Fig. 1A). The full list of methylation changes is accessible via Gene Expression Omnibus (http://www.ncbi.nlm.nih.gov/geo/; accession number GSE59065). The overall direction (hyper- or hypomethylation) of age-related methylation changes in CD4+ and CD8+ T cells was in strong correlation (r > 0.9) (Fig. 1B). Less than 2% of the age-related methylation sites in CD4+ and CD8+ T cells could be detected in PBLs, and majority of them were hypermethylated in older population. Nevertheless, all top sites with the highest methylation differences between younger and older individuals belonged to this group shared by CD4+, CD8+ T cells and PBLs (Fig. 1C).

### Increased methylome variation in aged CD8+ T cells

**Figure 2:**
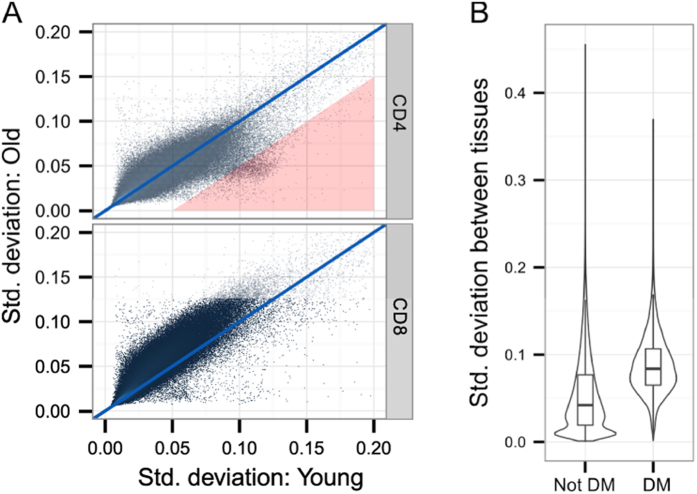
Methylation variability in T cells. (**A**) The scatter plots compare the standard deviation of every site within younger (x-axis) and older (y-axis) individuals. In CD4+ T cells (upper panel) there is a small subset of sites (marked with a red triangle) that is more variable among young subjects. This subset is enriched in genes involved in hemostasis according to g:Profiler Gene Ontology search (see Supplementary Table S2 for details). In CD8+ T cells (lower panel) majority of sites are more variable in older individuals. (**B**) Box/violin plots showing the variability of the differentially methylated (DM) and other sites (Not DM) in comparison with the methylation variability in a compendium of 17 different tissues. Note higher standard deviation value among differentially methylated sites.

The average methylation levels between CD4+ and CD8+ T cells from younger and older individuals were similar across different genomic locations (Supplementary Fig. S1A). However, given the high number of methylation changes and expansion of terminally differentiated CD8+ T cells with age, we expected the CD8+ T cell methylome to be more variable among the old age group. Indeed, we found increased age-related variability among majority of CpG sites in CD8+ T cells (Fig. 2A; note shift in methylome variability of CD8+ T cells from older individuals). This increased variation of CpG methylation in CD8+ T cells from older individuals was present in all gene and CpG island subregions (Supplementary Fig. S1B). On average, the CpG methylation variation levels in CD4+ T cells changed less with age, with the exception of a subset of CpG sites enriched for genes involved in wound healing according to Gene Ontology (GO) analysis (Fig. 2A, Supplementary Table S1). We then tested whether the CpG sites that were differentially methylated in CD8+ T cells of older individuals are in general more variable in somatic cells and compared our results with the methylomes from 17 human somatic tissues^28^. We found that the differentially methylated CpG sites had also higher variability in other tissues (Fig. 2B). Together the results showed that CD8+ T cells had higher age-related methylation variation than CD4+ T cells and that the majority of age-related changes in CD8+ T cells occur at CpG sites that are variably methylated in multiple tissues.

### Age-related hypermethylation occurs at CpG islands and regions with repressive histone marks

**Figure 3:**
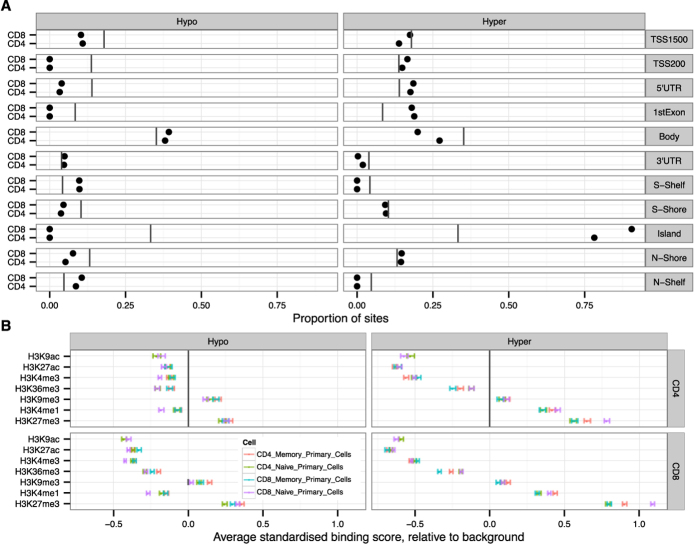
Location of differentially methylated sites in relation to gene subregions and histone modifications. (**A**) The proportions of differentially hypo- and hypermethylated sites that are related to various gene and CpG island subregions are marked with black dots. The vertical lines show the proportion of all CpG sites of HumanMethylation 450 K array located within each subregion. (**B**) The epigenetic landscape near the differentially methylated sites using Roadmap Epigenomics histone modification data from naïve and memory CD4+ and CD8+ cells. The figure shows the average difference in standardized binding score between differentially methylated and background of non-differentially methylated sites. The influence of gene and island subregion distribution for the sites (shown in Panel A) was subtracted before the analysis.

We found a large proportion (>80%) of the hypermethylated CpG sites located in CpG islands, while the hypomethylated CpG sites were more located at the borders of the CpG islands (CpG island shelves) and in gene bodies in both CD4+ and CD8+ T cells (Fig. 3A). Hypermethylation was also increased at gene regulatory (TSS200, 5´ UTR and 1^st^ exon) regions whereas hypomethylation was preferentially decreased in gene regulatory regions. This suggested that the hypermethylated regions are enriched in CpG islands located at 5´gene regulatory regions. We then analysed the histone modifications around the differentially methylated sites. To this end, we overlapped the differentially methylated sites in T cells with the reported locations of seven histone modifications in naïve and memory CD4+ and CD8+ T cells, available from the Roadmap Epigenomics project (Fig. 3B). The comparison showed similar distribution of histone marks in naïve and memory subsets of CD4+ and CD8+ T cells. Both age-related hypo- and hypermethylated regions were enriched in repressed chromatin marks (H3K27me3 and H3K9me3). In agreement, the differentially methylated sites had also lower levels of histone marks associated with active promoters (H3K9ac and H3K4me3), enhancers (H3K27ac) or exon regions (H3K36me3). However, another enhancer-associated modification, H3K4me1, which has been also associated with transposable elements^29^, was abundant at hypermethylated regions in older individuals. In summary, these results showed the enrichment of age-related hypermethylation at CpG islands and in regions with repressive histone marks.

### Age-related differential methylation occurs at repressed genes but in a subset of genes correlates inversely with expression

**Figure 4:**
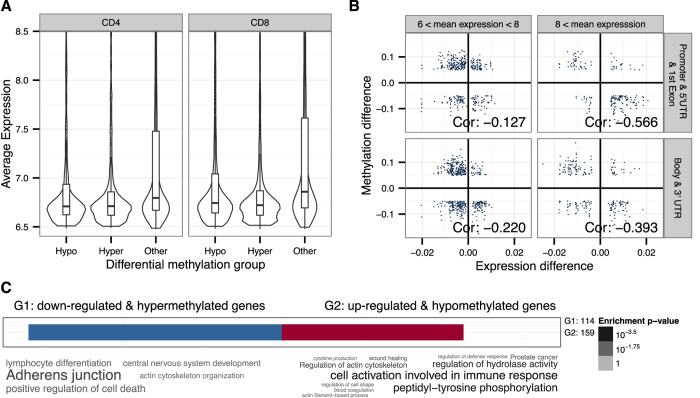
Gene expression and its relation to differential methylation. (**A**) The average log2 transformed expression levels of genes in CD4+ and CD8+ T cells are compared among the genes with decreased (Hypo), increased (Hyper) and unchanged (Other) methylation during ageing. The gene expression values lower than 6.5 are not included as they correspond to non-detectable gene expression. (**B**) The correlation between differentially expressed genes (x-axis) and corresponding differentially methylated sites (y-axis) in CD8+ T cells. Each dot corresponds to a CpG site-gene pair. The subplots are defined based on the average expression level and the location of the CpG site. (**C**) Functional annotation of genes (from panel B) with inverse correlation between their expression and the methylation of nearby CpG sites as word clouds. G1 corresponds to the group of genes with downregulated expression and hypermethylation and G2 corresponds to the group of genes with upregulated expression and hypomethylation (see Supplementary Table S2 for details).

To show that the age-related CpG methylation occurs near transcriptionally repressed genes, we analysed the expression profiles of the CD4+ and CD8+ T cells using the HumanHT-12 v4 Expression array. We compared the differentially methylated sites in respect to the average expression level of their nearest gene. Figure 4A shows that irrespective of the direction of methylation change, the differentially methylated sites were preferentially associated with genes having low or no expression.

We then studied the correlation of DNA methylation and expression in CD4+ and CD8+ T cells (Supplementary Table S2). Importantly, in a subset of genes that were expressed in CD8+ T cells (>log 6), we observed an inverse correlation between gene expression and DNA methylation (Fig. 4B). The inverse correlation was stronger among methylation sites that were located in gene regulatory and promoter regions and among genes with higher expression levels (>log 8). Functional annotation of the genes with the inverse correlation revealed their association with cell activation involved in immune response, tyrosine phosphorylation and lymphocyte differentiation among the hypomethylated genes (Fig. 4C, Supplementary Table S3). We then searched the promoter regions of the genes with inverse correlation of methylation and expression in CD8+ T cells for binding sites of transcription factors, which could be involved in the recruitment of DNA methylation modifier enzymes. We found a significant overrepresentation of sites for C2H2 zinc finger family transcription factors KLF4, MZF1 and SP1 (Supplementary Table S3), regardless of the expression levels of the genes. Together, the expression analysis confirmed that the majority of the differentially methylated sites are located near genes with low transcriptional activity. However, among the genes that were differentially expressed during ageing, we found significant inverse correlation between methylation and gene expression.

### DNA methylation correlates with the expression of genes involved in immune response and lineage differentiation

**Figure 5:**
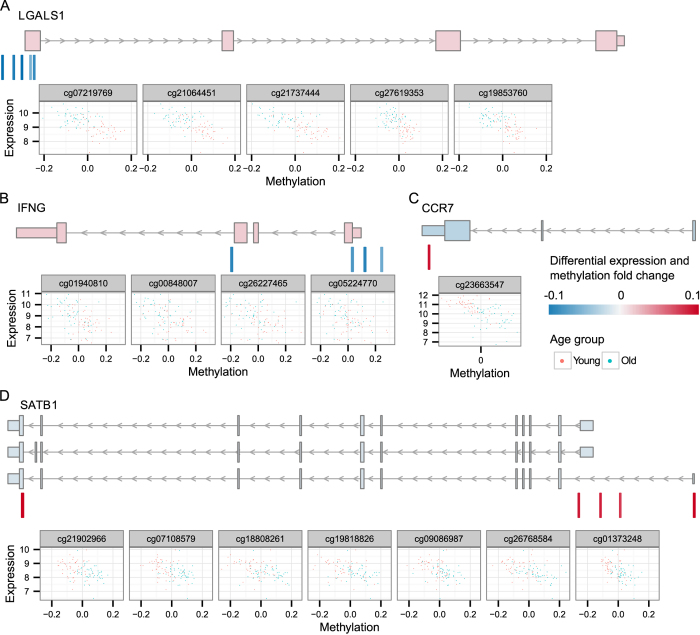
Examples of genes with known functional role in CD8+ T cells that display inverse correlation between gene expression and DNA methylation. (**A**) *LGALS1*, (**B**) *IFNG*, (**C**) *CCR7* and (**D**) *SATB1* gene. Each panel is composed of three sections. The top sections display the different transcripts of the gene, marked according to the expression level fold change between younger and older individuals. The middle sections show the associated CpG sites that are differentially methylated. The scatter plots in the bottom sections illustrate the correspondence between expression and methylation levels for each site separately. The scatter plots are displayed in the same order as the sites in the middle section; the red and blue dots display levels detected in the younger and older individuals, respectively.

To assess the potential functional effect of DNA methylation changes in aged T cells, we focused on the genes with inverse correlation between methylation and expression: 10 genes in CD4+ and 272 genes in CD8+ T cells (Supplementary Table S2). We found strong inverse correlation with the *LGALS1* gene encoding galectin 1, which is known to have a strong suppressive effect on T cell mediated immune responses due to its activity to induce apoptosis of activated T cells^30^. The increased expression of *LGALS1* with decreased methylation at its promoter region was present in both aged CD8+ and CD4+ T cells (Fig. 5). The other known genes with decreased methylation and increased expression in aged CD8+ T cells were the proinflammatory mediators *IFNG* and *CCL5*, as well as the cytolysis enzyme granzyme *GZMH* involved in CD8+ T cells effector functions (Supplementary Fig. S2). By contrast, older individuals showed increased methylation and decreased expression of the chemokine receptor *CCR7* responsible for T cell homing to lymph nodes and activation^31^, the membrane surface marker *CD27* involved in T cell expansion and induction of long-term memory^32^,^33^ and CD248 which regulates the proliferation of T cells^34^. Furthermore, we observed negative correlation for several master transcriptional regulators of the T cell lineage such as *SATB1*, a chromatin organizer in T cells, *TCF7*, a T cell specific factor that induces the T cell differentiation program and controls the expression of the CD3E molecule, as well as *BCL11B* and *RUNX3*, the crucial regulators of the T cell lineage. Among other top genes with inverse correlation between methylation and gene expression we found RGS10, a regulator of G-protein signalling acting as an inhibitor of T cell adhesion and KLRD1/CD94, a receptor for the HLA-E molecules. To confirm our array results, we verified the correlations for several genes (*IFNG, GZMH, CCR7, CD27, CD248,* and *SATB1*) using the Sequenom MassARRAY and real-time quantitative PCR in a smaller subset of samples from younger and older persons (Supplementary Fig. S3).

## Discussion

We here report age-related methylation changes that were identified in CD4+ and CD8+ T cells by analyzing more than 400,000 CpG sites from a total of 100 individuals.

The tissue-specificity of age-related DNA methylation has been reported previously^35^, yet several genome-wide studies have demonstrated similar, but not identical, age-related methylation changes in different tissues^36^,^37^,^38^,^39^. When we applied normalization based on measured granulocyte, monocyte and lymphocyte counts in our donor individuals to the blood DNA methylation profiles, the number of differential methylation sites in PBL samples decreased remarkably, indicating that the blood cell proportions that differ between individuals have impact on overall analysis of DNA methylation. However, the most significant methylation changes remained - they were shared by PBL and T cell samples and cannot be explained by the variation of blood cell proportions. This finding is in agreement with the recent study showing that most prominent epigenetic changes identified in blood cells retain their significance even after the analysis is adjusted for shifts in blood cell subtypes^21^. The top age-related DNA methylation changes found in any leukocyte subclass could accumulate in precursor cells at earlier stages of differentiation, for example during haematopoiesis, or reflect more general phenomenon of epigenetic drift with time. Nevertheless, our data show that although both CD4+ and CD8+ T cells share age-related methylation changes with PBL, many additional DNA methylation changes specific to T cells occur with age.

It should be noted that thymic involution influences T cell population during ageing^40^. T cells from older persons tend to have decreased percentages of naive cells and increased proportion of memory cells, which in CD8+ T cell compartment accumulate as terminally differentiated effector memory cells^41^. One of the limitations of our study is that the proportions of the naïve and memory cells differ between young and old people, and this could partly explain some of the methylation changes seen in our analysis. In agreement with the oligoclonal expansion and accumulation of terminally differentiated CD8+ cells, we found higher number of DNA methylation changes and increased methylation variation in aged CD8+ T cells in comparison to CD4+ cells. Whether the increased differential methylation is associated with the proliferation of CD8+ T cells in response to chronic CMV infection needs further studies.

Majority of the hypermethylated CpG sites were located in CpG islands, at silent gene promoter regions and were enriched for repressive marks such as H3K27me3, confirming the earlier reported links between age-related hypermethylation, gene inactivation and chromatin condensation^42^. Indeed, majority of age-related methylation changes seem not to affect the expression of nearby-positioned genes. However, in a subset of genes expressed in CD8+ T cells, we found a negative correlation between DNA methylation and transcription levels. Among those we identified genes with critical roles in T cell mediated immune responses. We found decreased methylation and increased expression of galectin 1 gene (*LGALS1*) that has multiple functions in suppressing immune responses, including controlling of T cell survival, TCR mediated signalling, regulatory T cell function, and by inhibiting anti-tumor T cell responses^30^,^43^,^44^. In addition, we found in older people the hypomethylation at the *IFNG* promoter, which correlated with the higher expression of the gene in their CD8+ T cells. Previous studies have shown high production of IFNγ the major proinflammatory cytokine, by activated CD8+ T cells after the stimulation by CMV antigens^10^,^45^. Decreased levels of DNA methylation and H3K4me3 repressive marks have been found at *Ifng* gene after the activation and differentiation of mouse CD8+ T cells in response to infections^46^. We also found hypomethylation and increased expression of the proinflammatory chemokine CCL5, the plasma levels of which are known to increase with age^10^, and of the GZMH gene, which is upregulated in effector T cells during infections and in chronic inflammatory diseases^47^. Demethylation of the *Ifng*, *Ccl5* and granzyme genes occurs during viral infection-induced differentiation of mouse effector and memory CD8+ T cells^48^,^49^. Furthermore, our finding of promoter hypermethylation of costimulatory CD27 and chemokine CCR7 receptor genes is in agreement with their downregulation in terminally differentiated anergic CD8+ T cells observed in aged individuals^41^. In this light, it is tempting to speculate that age-related chronic viral infections, such as CMV, may induce extensive oligoclonal proliferation of CD8+ T cells and result in changed DNA methylation profiles at genes involved in T cell responses to viral infections and in chronic inflammation.

T cell differentiation programme to specialized effector cells is guided by the action of several distinct transcription regulators. In CD8+ T cells, we identified age-related hypermethylation at several transcriptional regulator genes required for T cell lineage differentiation. SATB1, the T lineage-enriched global chromatin organizer, has important roles in T cell development and proliferation and ensures proper development of the lineage^50^,^51^. Furthermore, three other genes regulating T cell differentiation, *TCF7*, *BCL11B* and *RUNX3*^52^,^53^,^54^,^55^ were hypermethylated and had lower expression level in CD8+ T cells from older individuals. The expression of *TCF7*, *BCL11B* and *RUNX3* genes is required for the mature CD8+ T cell differentiation and is decreased with the acquisition of effector cell phenotype^56^,^57^. Our data thus show that ageing is associated with decreased expression and DNA hypermethylation of central T cell specific transcriptional regulator genes with fundamental roles in CD8+ T cell differentiation. Together, our results support the concept that the silencing of transcriptional regulator genes by DNA hypermethylation during ageing directs the gene expression profile towards the terminally differentiated effector CD8+ T cells.

In conclusion, although epigenome-wide studies with PBL have identified genes with methylation changes associated with age, the purification of cell subtypes allows more precise investigation of changes relevant to the altered function of specific cells. Our study shows that most of the gains in methylation that occur in ageing T cells are in transcriptionally repressed genes. However, the DNA methylation changes that are accompanied with gene expression changes affect many genes that are essential for the differentiation and function of T cells and shed light on the possible causes of the age-related decline in immune response. In addition, our study forms the basis to further evaluate the potential use of the identified DNA methylation changes as clinical markers of immunosenescence in older individuals.

## Materials and Methods

### Ethics statement

The study was approved by the Ethics Review Committee of Human Research of the University of Tartu, Estonia (permission no 206/T-4, date of issue 25.08.2011) and it was carried out in compliance with the Helsinki Declaration. All of the participants were older than 18 and a written informed consent to participate in the study was obtained from each individual prior to recruitment. All participants were healthy donors of the Estonian Genome Center of the University of Tartu. All methods were carried out in accordance with approved guidelines.

### Purification of cell populations

Peripheral blood mononuclear cells (PBMC) were extracted using Ficoll-Paque (GE Healthcare) gradient centrifugation. CD8+ T-cells and CD4+ T-cells were extracted from PBMCs by consecutive positive separation using microbeads (CD4+ #130-045-101; CD8+ #130-045-201) and AutoMACS technology (Miltenyi Biotec) according to the manufacturer’s protocol. The purity of extracted CD4+ T-cells was 91–95% and for CD8+ T-cells 88–91%. All cell populations were collected and stored as cell pellets in a −80 °C freezer.

### DNA extraction, bisulfite treatment and DNA methylation measurement

Genomic DNA was isolated from the purified cell pellets using the QIAmp DNA Mini Kit (Qiagen) according to the manufacturer’s protocol. DNA from whole blood was extracted by the salting-out method using 10 M ammonium acetate. The DNA was precipitated in isopropanol, washed in 70% ethanol, and finally resuspended in 1X TE buffer. The purity and concentrations of the DNA samples were measured by NanoDrop ND-1000 spectrophotometry. 500 ng of genomic DNA was treated with sodium bisulfite using the EZ DNA Methylation Kit (Zymo Research Corporation) according to the manufacturer’s instructions. DNA methylation analysis was performed using the Infinium Human Methylation 450 K BeadChip (Illumina). Quantitative analysis of DNA methylation was verified using Sequenom’s EpiTYPER T Complete Reagent Set and MassARRAY Analyzer 4 (Sequenom). DNA was amplified using bisulfite-converted DNA, Hot Start DNA Polymerase (Solis BioDyne) and specific primers, according to the EpiTYPER protocol. Primers were designed with Sequenom’s EpiDesigner program and are listed in Supplementary Table S4.

### RNA extraction, labelling, hybridization, and qPCR

RNA was extracted from the purified T-cells using the miRNeasy Mini Kit combined with recommended RNase-free DNase I treatment (both from Qiagen). RNA from whole blood was purified using the MagMAX™ for Stabilized Blood Tubes RNA Isolation Kit. RNA was concentrated using the Heraeus vacuum centrifugation system without heating. RNA was labeled and amplified using the TargetAmp-Nano Labeling Kit for Illumina Expression BeadChip (Epicentre Biotechnologies) with SuperScript III Reverse Transcriptase (Life Technologies), followed by purification with the RNeasy MinElute Cleanup Kit (Qiagen). RNA quality was assessed after extraction and after labelling using an Agilent 2100 Bioanalyzer and Agilent RNA 6000 Nano Kit (all from Agilent Technologies). Labeled RNA was hybridized to the HumanHT-12 v4 Expression BeadChip (Illumina) according to the manufacturer’s instructions. Verification of the expression of changed genes in CD4+ and CD8+ T cells was performed by qPCR using primers listed in Supplementary Table S4.

### Normalization of methylation data

Data pre-processing and quality control analyses were performed in R with the Bioconductor package *minfi,* using the original IDAT files extracted from the HiScanSQ scanner. ‘Raw’ pre-processing was used to convert the intensities from the red and the green channels into methylated and unmethylated signals. Beta values were computed using Illumina’s formula [beta = M/(M + U + 100)]. The difference in the distribution of beta values for type I and type II probes was corrected using “SWAN”^58^, a normalization method to address systematic changes between type I and type II probes. Detection p-values were obtained for every CpG probe in each sample. Failed positions were defined as signal levels lower than background from both the methylated and unmethylated channels.

Samples with detection p-value > 0.01 in more than 10% of the CpG sites and possibly contaminated or mixed up samples (determined using the 65 SNPs present on the methylation beadchip) were discarded. We mapped all the probe sequences to the human genome (build hg19) and discarded all probes that did not map uniquely, mapped to CpG sites with SNPs up to 3 bp upstream of the site, or mapped to the X/Y chromosomes. After data preprocessing, the total number of samples left for analysis was 296 (97+99+100). The total number of retained CpG sites was 399,653. The full data is stored at Gene Expression Omnibus (http://www.ncbi.nlm.nih.gov/geo/) with accession number GSE59065.

### Differential methylation analysis

Before performing differential methylation analysis the effects of known confounding variables were removed. For samples from CD4+ and CD8+ cells the effect of sex and array was corrected for. In the case of PBLs the levels of erythrocytes, platelets, monocytes, lymphocytes and granulocytes that were measured by blood biochemistry were additionally included as covariates. The correction was performed by fitting a linear model with these covariates to every CpG site and using the residuals for further analysis. The differential methylation analysis of younger and older persons was performed using moderated t-test from the Bioconductor limma package^59^. CpG sites with an average methylation difference >0.05 between the younger and the older subjects, and false discovery rate (FDR) <0.05 were considered differentially methylated.

### Differential expression analysis

Differentially expressed genes were identified using a moderated t-test from the Bioconductor limma package^59^. The same confounding factors used in the methylation analysis were applied for the different cell types. FDR <0.05 was used as threshold in all comparisons.

### Gene Ontology analysis

All Gene Ontology enrichment analyses were performed using the g:Profiler web toolkit^60^ and R package GOsummaries (http://www.bioconductor.org/packages/release/bioc/html/GOsummaries.html). To display less redundant and more specific results, GOsummaries uses several non-default filtering options in g:Profiler. First, we limited the number of genes in a category; the upper limit of the number of genes was set to 1000 and the lower limit was 50 genes. Second, the program uses a “Best per parent” hierarchical filtering option to remove categories that are closely related on a GO tree. Finally, the results from “Cellular Component” and “Molecular Function” sections of GO were removed, as too general and not informative. The statistical significance threshold was 0.05 for the false discovery rate provided by g:Profiler.

### Gene part overlaps

The CpG site associations with gene subregions were obtained from the annotation file provided by Illumina. In the file one site can be annotated to multiple regions if it is associated with more than one gene/island subregion.

### Histone modification overlaps

Roadmap Epigenomics^61^ histone modification data of naïve and memory CD4+ and CD8+ T cells was downloaded in Wig format from http://www.roadmapepigenomics.org/data . The read counts were extracted from the files for all differentially methylated and 20,000 non-differentially methylated CpG sites using the rtracklayer R package^62^. To make the scores comparable between modifications and cell types, these were log2 transformed (using pseudocount 1) and converted into z-scores based on the background distribution of the 20,000 sites. The coefficients and confidence intervals shown in Fig. 3 were obtained from a linear model that predicted the scores using the direction of differential methylation. In order to account for the preferential distribution of histone modifications along the genes and CpG islands, this information was also included in the linear models.

### Correlation between methylation and gene expression

The CpG sites were associated with genes based on annotated gene names (provided by Illumina). The gene associations to gene expression probes were obtained using gConvert tool of the g:Profiler web toolkit^60^. In the correlation plots differentially methylated/expressed genes were used (described above). The plots with gene transcripts were drawn using the Gviz package (http://www.bioconductor.org/packages/devel/bioc/html/Gviz.html), and were based on the known genes table from the UCSC genome browser, genome build hg19.

## Additional Information

**How to cite this article**: Tserel, L. *et al.* Age-related profiling of DNA methylation in CD8+ T cells reveals changes in immune response and transcriptional regulator genes. *Sci. Rep.* **5**, 13107; doi: 10.1038/srep13107 (2015).

## Supplementary Material

Supplementary Information
