# Supplementary material for: Age-related profiling of DNA methylation in CD8+ T cells reveals changes in immune response and transcriptional regulator genes
Source: Sci Rep. 2015 Aug 19;5:13107. doi: 10.1038/srep13107 (PMC4541364; doi:10.1038/srep13107)
Supplement: Supplementary Information [file srep13107-s1.pdf]

Supplementary information

Age-related profiling of DNA methylation in CD8+ T cells reveals changes in immune response and transcriptional regulator genes

*Liina Tserel[1], *Raivo Kolde[2], Maia Limbach[1], Konstantin Tretyakov[2], Silva Kasela[3,4], Kai Kisand[1], Mario Saare[1], Jaak Vilo[2], Andres Metspalu[3,4], Lili Milani[3†] and Pärt Peterson[1†]

Supplementary files

The following additional data are available with the online version of this paper.

Supplementary Figure S1 shows average levels and standard deviations of methylation in younger and older CD4+ and CD8+ T cells.

Supplementary Figure S2 shows genes with inverse correlation of methylation and expression of data from Human Methylation 450K and HumanHT-12v4 Expression arrays.

Supplementary Figure S3 shows the verification using the Sequenom MassARRAY platform and qPCR.

Supplementary Table S1 lists g:Profiler pathway analysis results in Figures 2A.

Supplementary Table S2A lists the differentially methylated genes with variable expression levels (and their correlation coefficients) in younger and older CD4+ T cells.

Supplementary Table S2B lists the differentially methylated genes with variable expression levels (and their correlation coefficients) in younger and older CD8+ T cells.

Supplementary Table S3 lists g:Profiler pathway analysis results in Figures 4C.

Supplementary Table S4 lists transcription factor binding sites in promoter regions of genes with inverse correlation between methylation and expression.

Supplementary Table S5 lists the oligonucleotides used for the Sequenom MassARRAY and qPCR experiments.

Figure S1. Average methylation and methylation variability in CD4+ and CD8+ T cells. (A) The average methylation divided according to CpG island and gene subregions. (B) Average standard deviation of methylation divided according to CpG island and gene subregions.

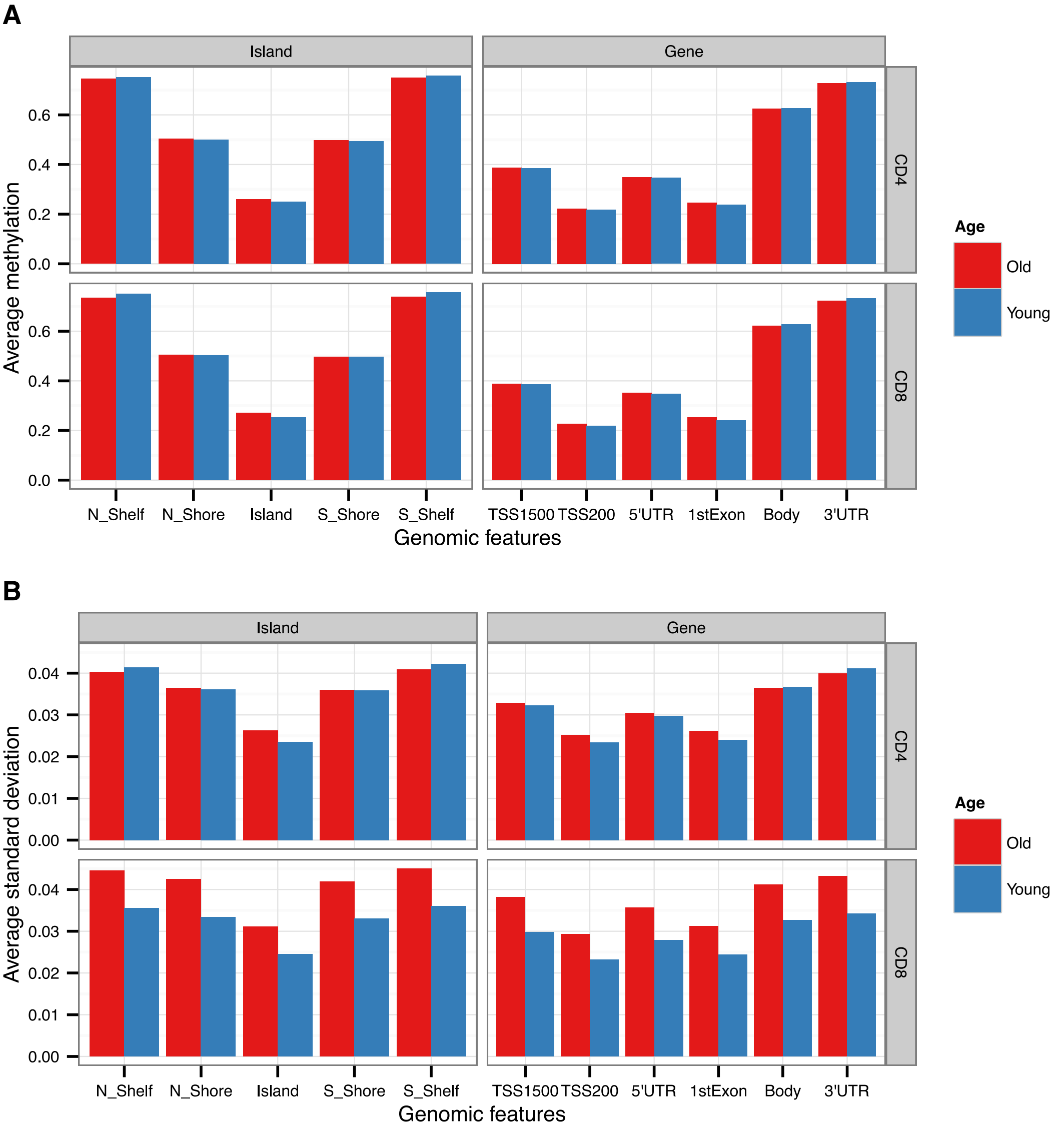

Figure S2. Genes with inverse correlation between gene expression and DNA methylation. The top sections display the transcripts of the gene. The transcripts are coloured according to their fold change of expression level between younger and older individuals. The middle sections show the positions of associated CpG sites that are differentially methylated. The scatter plots in the bottom sections illustrate the correspondence between expression and methylation levels for each site separately. The scatter plots are displayed in the same order as the sites in the middle section; the red and blue dots display levels detected in the younger and older individuals, respectively.

BCL11B

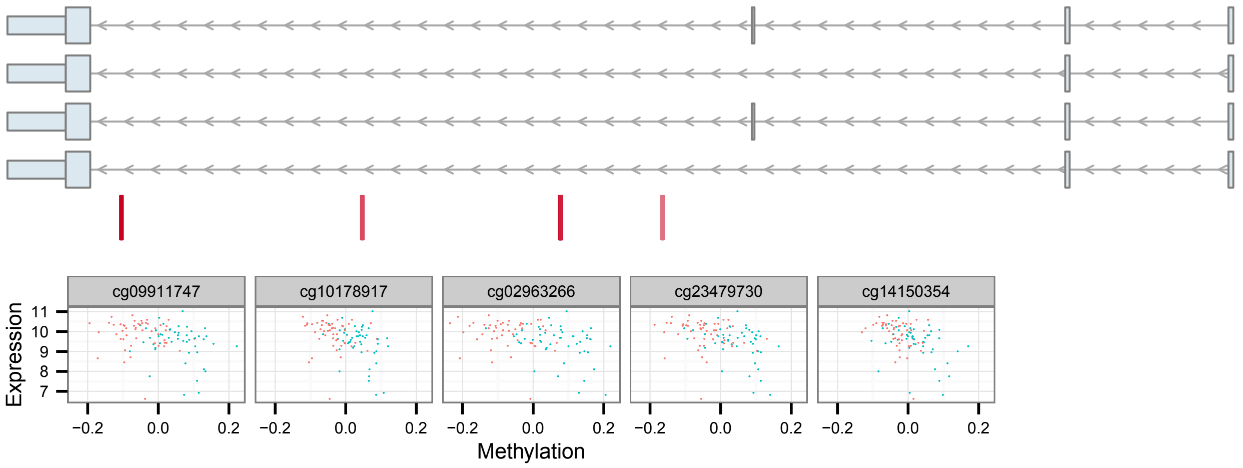

CCL5

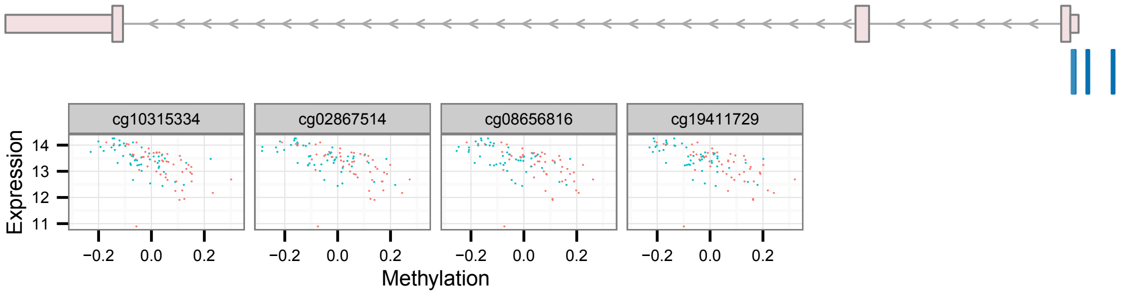

CD3E

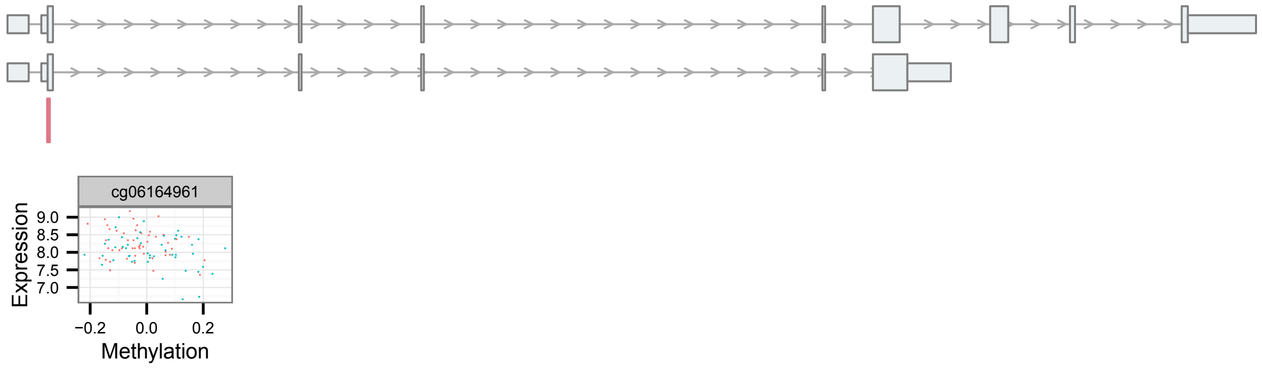

CD27

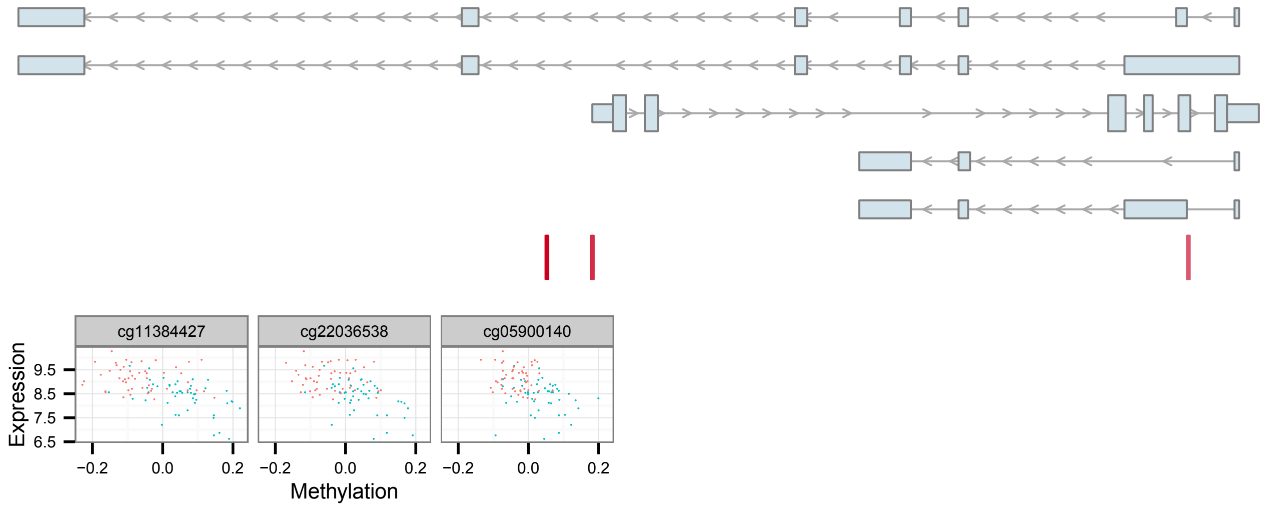

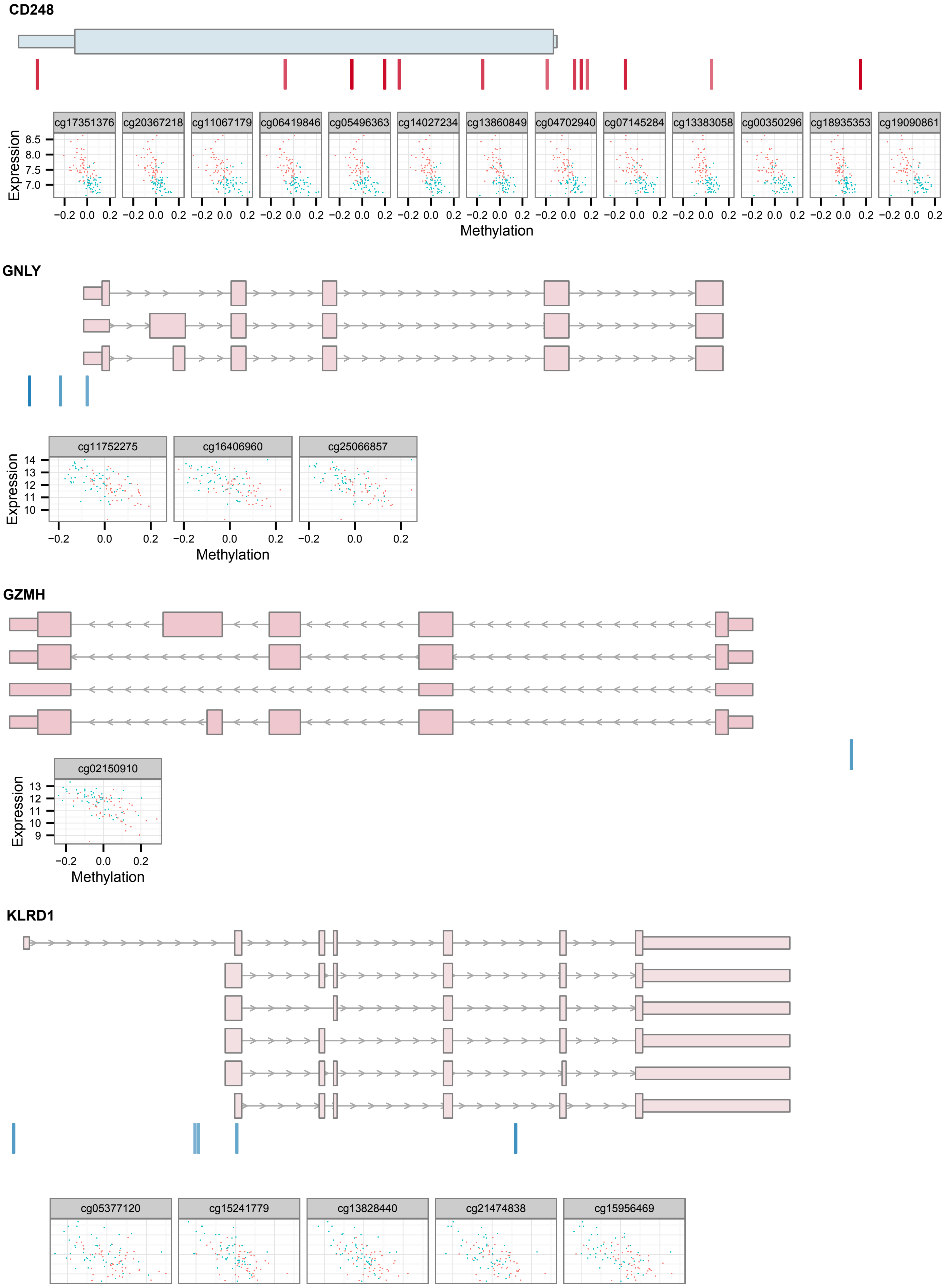
CD248
cg17351376
cg20367218
cg11067179
cg06419846
cg05496363
cg14027234
cg13860849
cg04702940
cg07145284
cg13383058
cg00350296
cg18935353
cg19090861
Expression
8.5
8.0
7.5
7.0
−0.2 0.0 0.2
Methylation
GNLY
cg11752275
cg16406960
cg25066857
Expression
14
13
12
11
10
−0.2 0.0 0.2
Methylation
GZMH
cg02150910
Expression
13
12
11
10
9
−0.2 0.0 0.2
Methylation
KLRD1
cg05377120
cg15241779
cg13828440
cg21474838
cg15956469

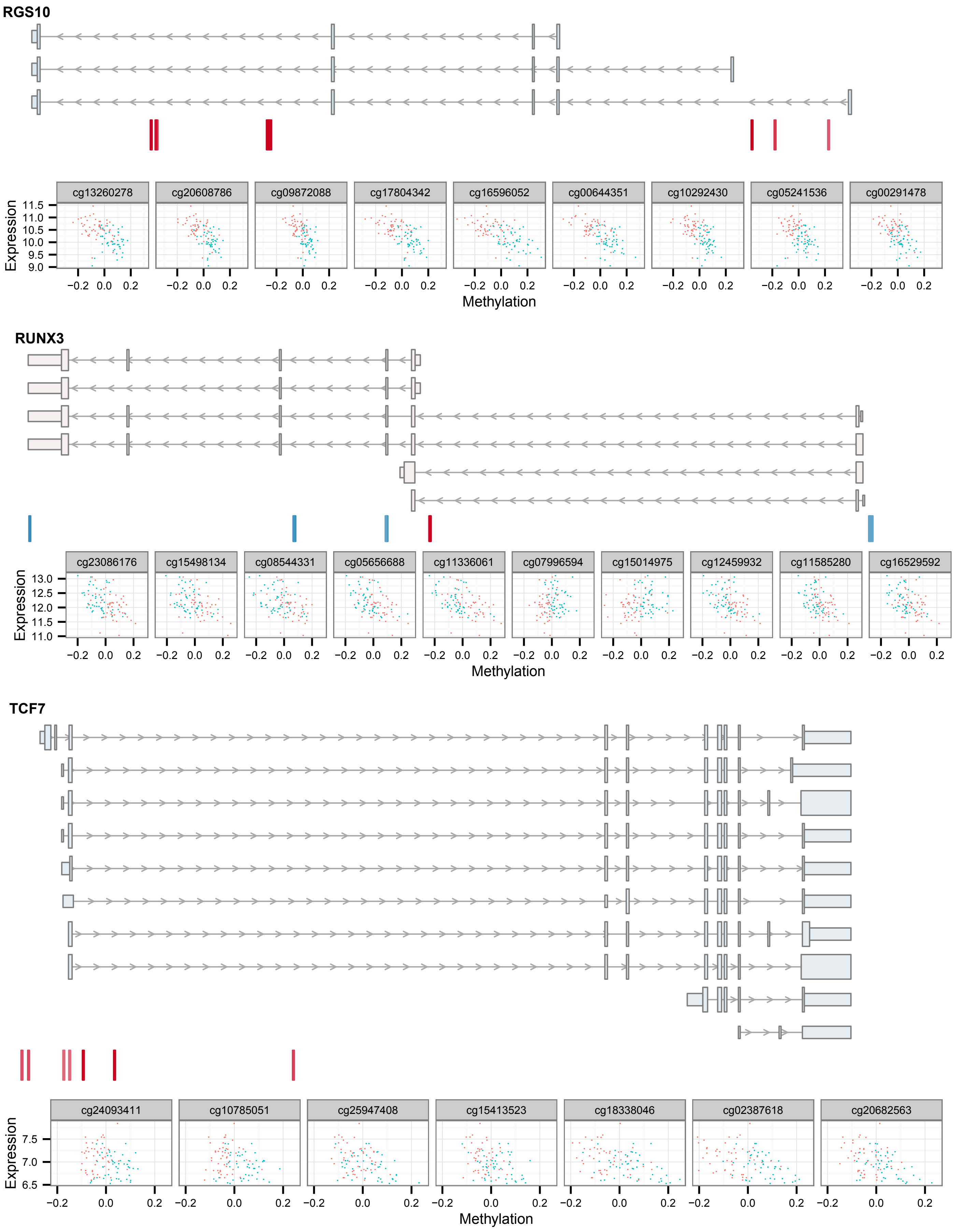
RGS10
cg13260278
cg20608786
cg09872088
cg17804342
cg16596052
cg00644351
cg10292430
cg05241536
cg00291478
Expression
11.5
11.0
10.5
10.0
9.5
9.0
−0.2 0.0 0.2
Methylation
RUNX3
cg23086176
cg15498134
cg08544331
cg05656688
cg11336061
cg07996594
cg15014975
cg12459932
cg11585280
cg16529592
Expression
13.0
12.5
12.0
11.5
11.0
−0.2 0.0 0.2
Methylation
TCF7
cg24093411
cg10785051
cg25947408
cg15413523
cg18338046
cg02387618
cg20682563
Expression
7.5
7.0
6.5
−0.2 0.0 0.2
Methylation

Figure S3. Verification of the inverse methylation and gene expression in a subgroup of genes from CD8+ T cells. To verify the age-related inverse correlation between methylation and gene expression Sequenom MassARRAY and qPCR analysis were used in a set of 14 young and 15 old subjects (IFNG, GZMH, CCR7 and CD27) or 16 young and 16 old subjects (CD248 and SATB1). Red dots represent the young and blue dots represent the old subjects, whereas sample number varies due to technical limitations of Sequenom MassARRAY. Correlation between methylation and gene expression was evaluated using the Spearman correlation coefficient. *P < 0.05, **P < 0.01 and ***P < 0.001.

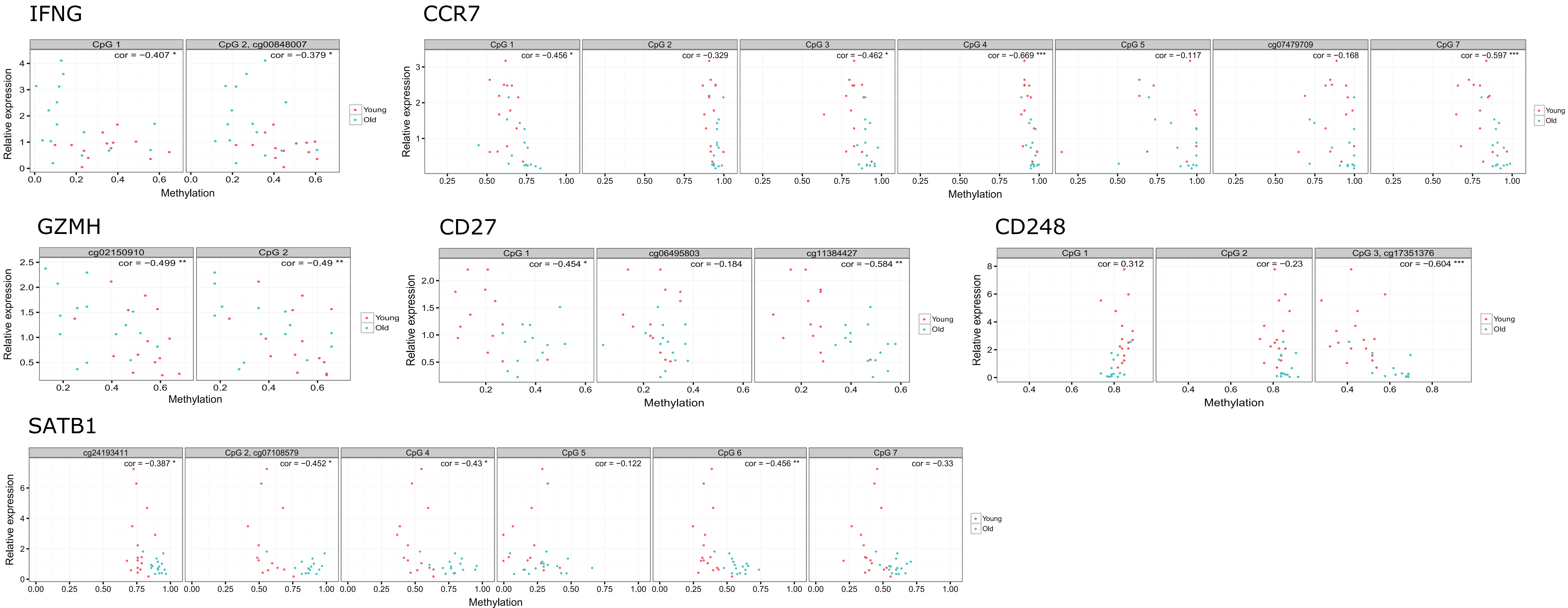

Supplementary Table 1. List of enriched functional terms sorted by p-value. The results were generated with g:Profiler and are graphically depicted in Fig. 2A.

	Query	p.value	term.s	query.	overla	precisio	recall	term	term.name
CD4	1,75E-10	696	1170	87	0,074	0,125	GO:0042060	16	wound healing
CD4	6,42E-10	568	1170	75	0,064	0,132	GO:0007599	47	hemostasis
CD4	1,08E-09	562	1170	74	0,063	0,132	GO:0007596	46	blood coagulation
CD4	1,62E-09	956	1166	105	0,09	0,11	GO:0001775	11	cell activation
CD4	4,52E-08	479	1159	63	0,054	0,132	GO:0006897	40	endocytosis
CD4	3,53E-07	994	1162	101	0,087	0,102	GO:0007243	50	intracellular protein kinase cascade
CD4	1,05E-06	955	1166	97	0,083	0,102	GO:0009967	36	positive regulation of signal transduction
CD4	4,39E-06	968	1098	92	0,084	0,095	GO:0080134	17	regulation of response to stress
CD4	2,28E-05	702	1159	74	0,064	0,105	GO:0007169	39	transmembrane receptor protein tyrosine kinase signaling pathway
CD4	2,96E-05	653	1159	70	0,06	0,107	GO:0070848	27	response to growth factor stimulus
CD4	4,42E-05	473	1172	56	0,048	0,118	GO:0097190	19	apoptotic signaling pathway
CD4	9,30E-05	259	1164	37	0,032	0,143	GO:0007015	22	actin filament organization
CD4	9,78E-05	254	1158	36	0,031	0,142	REAC:109582	26	Hemostasis
CD4	0,00035	623	1166	65	0,056	0,104	GO:0006954	6	inflammatory response
CD4	0,000453	126	981	21	0,021	0,167	GO:0042089	32	cytokine biosynthetic process
CD4	0,000542	577	1166	61	0,052	0,106	GO:0002764	15	immune response-regulating signaling pathway
CD4	0,000606	961	1175	89	0,076	0,093	GO:0016477	48	cell migration
CD4	0,00061	628	1175	65	0,055	0,104	GO:0048667	41	cell morphogenesis involved in neuron differentiation
CD4	0,000857	667	1164	67	0,058	0,1	GO:0042330	20	taxis
CD4	0,00123	512	1164	55	0,047	0,107	GO:0032101	29	regulation of response to external stimulus
CD4	0,00152	72	1130	16	0,014	0,222	GO:0045807	7	positive regulation of endocytosis
CD4	0,00161	58	1083	14	0,013	0,241	GO:0018210	45	peptidyl-threonine modification
CD4	0,00175	566	1175	59	0,05	0,104	GO:0007409	18	axonogenesis
CD4	0,00397	975	1175	87	0,074	0,089	GO:0042325	49	regulation of phosphorylation
CD4	0,00438	111	774	16	0,021	0,144	GO:0042476	21	odontogenesis
CD4	0,00531	571	1177	58	0,049	0,102	GO:0014070	25	response to organic cyclic compound
CD4	0,00629	725	1156	68	0,059	0,094	GO:0006812	31	cation transport
CD4	0,00633	262	1143	33	0,029	0,126	GO:0070482	51	response to oxygen levels
CD4	0,00644	715	1173	68	0,058	0,095	GO:0048534	4	hematopoietic or lymphoid organ development
CD4	0,00735	61	838	12	0,014	0,197	GO:0002367	23	cytokine production involved in immune response
CD4	0,00859	447	1175	48	0,041	0,107	GO:0007265	33	Ras protein signal transduction
CD4	0,01	794	1175	73	0,062	0,092	GO:0051094	2	positive regulation of developmental process

Supplementary Table S2A. Correlations of differentially methylated genes with expression levels in CD4+ T cells.

Gene	CpG site ID	Chr	Position	Methylation difference	Methylation P-value	Methylation FDR	Expression probe ID (ILMN_*)	Expression logFC	Expression P-value	Expression FDR	Methylation-expression correlation
FLNB	cg17338821	3	57 995 782	0,057	4,52E-14	1,17E-10	1664922	-0,009	3,68E-11	3,72E-07	-0,49
LGALS1	cg21737444	22	38 071 591	-0,059	4,73E-06	6,47E-05	1723978	0,010	1,51E-09	7,63E-06	-0,47
LGALS1	cg07219769	22	38 071 455	-0,054	6,96E-06	8,77E-05	1723978	0,010	1,51E-09	7,63E-06	-0,47
FLNB	cg23730027	3	57 995 180	0,070	2,41E-07	6,54E-06	1664922	-0,009	3,68E-11	3,72E-07	-0,39
NUCB2	cg02075820	11	17 300 909	0,055	1,07E-04	8,13E-04	1655913	-0,006	1,24E-06	2,18E-03	-0,38
ACTN1	cg15867698	14	69 438 267	0,067	2,49E-10	4,23E-08	2232177	-0,009	2,07E-05	1,79E-02	-0,37
INPP4B	cg18376497	4	143 488 622	-0,065	7,01E-09	4,72E-07	2198878	0,008	9,74E-07	1,79E-03	-0,36
PDE4D	cg03323696	5	59 189 120	0,085	5,77E-12	3,00E-09	1893697	-0,003	8,00E-05	4,91E-02	-0,23
FLNB	cg12194336	3	58 067 906	0,050	1,46E-05	1,60E-04	1664922	-0,009	3,68E-11	3,72E-07	-0,22
PDE4D	cg24628676	5	59 189 846	0,052	1,82E-09	1,77E-07	1893697	-0,003	8,00E-05	4,91E-02	-0,20
CTLA4	cg26091609	2	204 734 181	-0,054	2,59E-04	1,70E-03	1763487	0,008	5,03E-05	3,43E-02	-0,18
PDE4D	cg07190535	5	59 189 843	0,056	1,30E-11	5,29E-09	1893697	-0,003	8,00E-05	4,91E-02	-0,18
PDE4D	cg26078977	5	59 189 467	0,065	8,32E-11	1,95E-08	1893697	-0,003	8,00E-05	4,91E-02	-0,16
B3GALT5	cg26390598	21	41 032 396	-0,063	1,17E-06	2,18E-05	2378654	0,001	3,40E-05	2,55E-02	-0,14
CD70	cg11904429	19	6 592 554	-0,050	2,46E-03	1,13E-02	1760247	0,003	4,77E-06	5,68E-03	-0,12
ACTN1	cg00739471	14	69 415 588	0,059	1,90E-05	1,98E-04	2232177	-0,009	2,07E-05	1,79E-02	-0,10
AUTS2	cg14797899	7	69 882 555	-0,052	2,27E-08	1,12E-06	1749081	-0,006	4,94E-05	3,43E-02	-0,05
AUTS2	cg09703727	7	69 447 465	-0,062	4,14E-08	1,75E-06	1749081	-0,006	4,94E-05	3,43E-02	0,04
AUTS2	cg08903389	7	69 104 289	-0,074	2,42E-10	4,14E-08	1749081	-0,006	4,94E-05	3,43E-02	0,08
ZFHX3	cg05704496	16	72 993 237	-0,079	6,54E-08	2,47E-06	1808587	-0,002	5,19E-06	6,01E-03	0,09
PDE4D	cg27583655	5	59 126 518	-0,060	2,17E-05	2,19E-04	1893697	-0,003	8,00E-05	4,91E-02	0,12
PDE4D	cg21023952	5	59 783 035	-0,068	1,19E-07	3,84E-06	1893697	-0,003	8,00E-05	4,91E-02	0,13
AUTS2	cg10619644	7	69 149 951	-0,066	1,09E-11	4,70E-09	1749081	-0,006	4,94E-05	3,43E-02	0,16
PDE4D	cg07267294	5	58 893 722	-0,082	2,78E-10	4,58E-08	1893697	-0,003	8,00E-05	4,91E-02	0,16
KANK1	cg08677535	9	505 721	0,059	3,66E-10	5,53E-08	1773427	0,005	5,39E-05	3,57E-02	0,21
AUTS2	cg08474953	7	70 170 620	-0,050	2,20E-12	1,52E-09	1749081	-0,006	4,94E-05	3,43E-02	0,27
AFF3	cg13361307	2	100 365 075	-0,054	4,01E-06	5,68E-05	1775235	-0,004	8,09E-10	4,68E-06	0,27
CNTNAP1	cg21692620	17	40 835 849	0,075	2,64E-12	1,74E-09	1692398	0,005	5,43E-06	6,10E-03	0,29
AFF3	cg14793844	2	100 516 880	-0,072	1,36E-06	2,45E-05	1775235	-0,004	8,09E-10	4,68E-06	0,30
NRCAM	cg24429533	7	107 886 896	-0,052	7,22E-06	9,04E-05	1722809	-0,003	9,61E-09	4,24E-05	0,31
CACHD1	cg25210796	1	65 089 808	-0,058	4,07E-06	5,74E-05	1738854	-0,005	3,46E-13	1,40E-08	0,33
MMP28	cg11992940	17	34 094 454	-0,057	6,76E-08	2,53E-06	2399016	-0,004	1,06E-05	1,04E-02	0,33
PAM	cg10640944	5	102 201 890	0,089	3,40E-10	5,27E-08	2313901	0,006	3,43E-06	4,34E-03	0,34
CACHD1	cg24639703	1	64 986 155	-0,067	7,15E-06	8,97E-05	1738854	-0,005	3,46E-13	1,40E-08	0,35
NRCAM	cg24429533	7	107 886 896	-0,052	7,22E-06	9,04E-05	2411236	-0,007	4,18E-12	5,64E-08	0,36
PAM	cg24531022	5	102 201 825	0,095	7,55E-13	7,62E-10	2313901	0,006	3,43E-06	4,34E-03	0,38
CACHD1	cg11795488	1	65 038 492	-0,057	6,19E-06	8,01E-05	1738854	-0,005	3,46E-13	1,40E-08	0,42

Supplementary Table 2B. Correlations of differentially methylated genes with variable expression levels in CD8+ T cells. Only correlations >0.30 or <-0.30 are shown.

Gene	CpG site ID	Chr	Position	Methylation difference	Methylation P-value	Methylation FDR	Expression probe ID (ILMN_*)	Expression logFC	Expression P-value	Expression FDR	Methylation-expression correlation
LGALS1	cg19853760	22	38 071 677	-0,080	1,46E-13	2,15E-11	1723978	0,016	2,12E-12	6,14E-09	-0,64
LGALS1	cg21064451	22	38 071 534	-0,088	5,97E-12	2,97E-10	1723978	0,016	2,12E-12	6,14E-09	-0,64
FLNB	cg17338821	3	57 995 782	0,059	4,05E-09	5,98E-08	1664922	-0,013	3,39E-12	9,15E-09	-0,64
LGALS1	cg21737444	22	38 071 591	-0,100	3,74E-10	8,25E-09	1723978	0,016	2,12E-12	6,14E-09	-0,63
RGS10	cg17804342	10	121 271 663	0,135	5,08E-16	9,57E-13	1733538	-0,011	2,26E-11	3,92E-08	-0,62
ACTN1	cg04958055	14	69 404 437	0,054	1,02E-04	3,96E-04	2232177	-0,017	1,94E-07	6,35E-05	-0,62
CD248	cg17351376	11	66 082 046	0,081	8,97E-12	4,07E-10	1726589	-0,012	5,95E-15	4,81E-11	-0,62
RGS10	cg16596052	10	121 271 746	0,176	7,69E-16	1,14E-12	1733538	-0,011	2,26E-11	3,92E-08	-0,62
KLRD1	cg13828440	12	10 459 972	-0,053	2,29E-03	6,66E-03	1799134	0,011	2,14E-04	1,63E-02	-0,62
CCR7	cg23663547	17	38 710 320	0,092	1,31E-11	5,43E-10	1715131	-0,025	7,90E-15	5,33E-11	-0,62
RGS10	cg00644351	10	121 271 840	0,130	4,74E-13	4,75E-11	1733538	-0,011	2,26E-11	3,92E-08	-0,62
RGS10	cg20608786	10	121 265 838	0,093	1,14E-12	8,78E-11	1733538	-0,011	2,26E-11	3,92E-08	-0,61
CCL5	cg19411729	17	34 207 663	-0,105	4,41E-06	2,35E-05	2098126	0,010	9,37E-06	1,54E-03	-0,61
KLRD1	cg15241779	12	10 459 912	-0,051	5,08E-03	1,36E-02	1799134	0,011	2,14E-04	1,63E-02	-0,61
GNLY	cg11752275	2	85 921 036	-0,088	2,63E-08	2,86E-07	1708779	0,012	1,05E-03	4,82E-02	-0,61
KLRD1	cg15241779	12	10 459 912	-0,051	5,08E-03	1,36E-02	1797988	0,010	2,83E-05	3,65E-03	-0,59
KLRD1	cg13828440	12	10 459 972	-0,053	2,29E-03	6,66E-03	1797988	0,010	2,83E-05	3,65E-03	-0,59
LGALS1	cg07219769	22	38 071 455	-0,100	5,01E-13	4,92E-11	1723978	0,016	2,12E-12	6,14E-09	-0,59
CCL5	cg10315334	17	34 207 332	-0,081	3,37E-05	1,45E-04	2098126	0,010	9,37E-06	1,54E-03	-0,59
KLRD1	cg15956469	12	10 465 272	-0,075	3,73E-04	1,28E-03	1799134	0,011	2,14E-04	1,63E-02	-0,59
CCL5	cg02867514	17	34 207 341	-0,104	9,00E-06	4,44E-05	2098126	0,010	9,37E-06	1,54E-03	-0,59
SPINT2	cg27176870	19	38 754 930	0,065	7,29E-10	1,43E-08	1800739	-0,011	3,68E-08	1,78E-05	-0,59
KLRD1	cg15956469	12	10 465 272	-0,075	3,73E-04	1,28E-03	1797988	0,010	2,83E-05	3,65E-03	-0,58
CD248	cg18935353	11	66 085 250	0,055	7,28E-11	2,15E-09	1726589	-0,012	5,95E-15	4,81E-11	-0,58
GNLY	cg16406960	2	85 921 252	-0,066	3,56E-04	1,23E-03	1708779	0,012	1,05E-03	4,82E-02	-0,58
CD248	cg06419846	11	66 083 697	0,094	1,72E-12	1,18E-10	1726589	-0,012	5,95E-15	4,81E-11	-0,58
NMUR1	cg03164561	2	232 396 386	-0,057	6,62E-04	2,16E-03	1729287	0,002	1,08E-03	4,91E-02	-0,58
ABLIM1	cg14608903	10	116 287 249	0,073	7,45E-09	9,97E-08	1785424	-0,018	9,63E-12	1,95E-08	-0,58
KLRD1	cg21474838	12	10 460 612	-0,059	1,49E-03	4,51E-03	1799134	0,011	2,14E-04	1,63E-02	-0,58
NMUR1	cg24430754	2	232 396 241	-0,083	1,53E-05	7,13E-05	1729287	0,002	1,08E-03	4,91E-02	-0,58
NRCAM	cg06570167	7	108 095 719	0,057	1,09E-09	2,01E-08	2411236	-0,020	2,37E-21	9,60E-17	-0,57
CD248	cg20367218	11	66 083 224	0,068	3,33E-11	1,14E-09	1726589	-0,012	5,95E-15	4,81E-11	-0,57
APOBEC3H	cg24424889	22	39 494 175	-0,093	2,39E-06	1,37E-05	1664828	0,007	1,36E-05	2,09E-03	-0,57
PATL2	cg01647795	15	44 969 244	-0,076	8,31E-06	4,14E-05	1768551	0,014	6,76E-09	4,97E-06	-0,57
GNLY	cg11752275	2	85 921 036	-0,088	2,63E-08	2,86E-07	1790692	0,015	2,19E-04	1,66E-02	-0,57
RGS10	cg00291478	10	121 301 041	0,064	1,72E-06	1,02E-05	1733538	-0,011	2,26E-11	3,92E-08	-0,57
GZMH	cg02150910	14	25 079 357	-0,068	9,56E-04	3,02E-03	1731233	0,021	7,97E-08	3,16E-05	-0,57
RGS10	cg10292430	10	121 297 031	0,101	9,06E-16	1,24E-12	1733538	-0,011	2,26E-11	3,92E-08	-0,57
PCSK5	cg13670316	9	78 507 533	0,080	1,25E-11	5,25E-10	1767934	-0,012	1,52E-13	7,67E-10	-0,57

ACTN1	cg08152926	14	69 446 685	0,070	5,03E-08	4,92E-07	2232177	-0,017	1,94E-07	6,35E-05	-0,56
CCL5	cg08656816	17	34 207 454	-0,115	3,69E-07	2,68E-06	2098126	0,010	9,37E-06	1,54E-03	-0,56
CHMP7	cg16193207	8	23 107 961	0,110	1,82E-13	2,48E-11	1795933	-0,010	6,94E-09	4,97E-06	-0,56
TXK	cg17984638	4	48 136 452	0,106	1,43E-08	1,72E-07	1741143	-0,013	3,56E-10	4,50E-07	-0,56
CD248	cg00350296	11	66 084 841	0,086	2,53E-08	2,77E-07	1726589	-0,012	5,95E-15	4,81E-11	-0,56
CD248	cg14027234	11	66 084 163	0,074	4,81E-12	2,52E-10	1726589	-0,012	5,95E-15	4,81E-11	-0,56
ADRB2	cg08370787	5	148 208 041	-0,069	2,72E-06	1,53E-05	1695590	0,009	1,01E-05	1,63E-03	-0,56
CDK5R1	cg19634527	17	30 814 814	0,095	3,81E-08	3,91E-07	1730928	-0,009	2,07E-07	6,71E-05	-0,56
LGALS1	cg27619353	22	38 071 651	-0,051	1,70E-10	4,28E-09	1723978	0,016	2,12E-12	6,14E-09	-0,55
RGS10	cg09872088	10	121 265 893	0,072	1,64E-14	5,72E-12	1733538	-0,011	2,26E-11	3,92E-08	-0,55
IFT140	cg08006309	16	1 587 810	0,114	5,61E-12	2,83E-10	1751559	-0,007	1,40E-07	5,07E-05	-0,55
NKG7	cg19564512	19	51 874 939	-0,059	4,23E-04	1,44E-03	1682993	0,012	7,53E-05	7,23E-03	-0,55
PPP2R2B	cg15021031	5	146 333 034	-0,061	7,96E-06	3,98E-05	1660732	0,010	2,02E-04	1,57E-02	-0,55
KLRD1	cg21474838	12	10 460 612	-0,059	1,49E-03	4,51E-03	1797988	0,010	2,83E-05	3,65E-03	-0,55
CD248	cg19090861	11	66 085 958	0,100	2,08E-10	5,07E-09	1726589	-0,012	5,95E-15	4,81E-11	-0,55
ZBED3	cg19657875	5	76 382 381	0,078	6,64E-12	3,22E-10	1915076	-0,008	1,53E-09	1,44E-06	-0,55
TTC38	cg04431254	22	46 684 772	-0,057	1,70E-07	1,38E-06	2059886	0,012	9,05E-07	2,27E-04	-0,55
LRRN3	cg09837977	7	110 731 201	0,108	3,16E-14	8,36E-12	2048591	-0,009	7,67E-11	1,11E-07	-0,54
CD248	cg11067179	11	66 083 541	0,114	9,95E-13	7,95E-11	1726589	-0,012	5,95E-15	4,81E-11	-0,54
TXK	cg02600394	4	48 136 234	0,088	1,46E-09	2,56E-08	1741143	-0,013	3,56E-10	4,50E-07	-0,54
SPINT2	cg23090870	19	38 755 753	0,079	2,62E-13	3,16E-11	1800739	-0,011	3,68E-08	1,78E-05	-0,54
NKG7	cg12916723	19	51 876 788	-0,063	4,08E-05	1,73E-04	1682993	0,012	7,53E-05	7,23E-03	-0,54
ANXA2	cg02072495	15	60 689 285	-0,127	1,52E-09	2,65E-08	2409167	0,011	1,14E-07	4,25E-05	-0,54
CD27	cg11384427	12	6 553 585	0,119	2,64E-10	6,17E-09	1688959	-0,014	8,39E-09	5,66E-06	-0,54
PDE9A	cg08729135	21	44 073 555	0,052	5,44E-08	5,26E-07	2306540	-0,015	1,61E-11	2,96E-08	-0,54
FAM134B	cg00461022	5	16 618 052	0,115	1,18E-13	1,88E-11	2387952	-0,012	2,23E-09	2,00E-06	-0,54
ACTN1	cg13693582	14	69 441 902	0,086	2,65E-10	6,18E-09	2232177	-0,017	1,94E-07	6,35E-05	-0,53
CD248	cg05496363	11	66 083 766	0,078	5,30E-10	1,10E-08	1726589	-0,012	5,95E-15	4,81E-11	-0,53
RASSF1	cg19854901	3	50 376 216	-0,117	1,45E-10	3,76E-09	1734205	0,006	1,11E-03	4,99E-02	-0,53
SATB1	cg01373248	3	18 480 297	0,065	1,19E-09	2,16E-08	3247857	-0,007	7,00E-09	4,97E-06	-0,53
TXK	cg20981615	4	48 136 280	0,104	2,53E-09	4,04E-08	1741143	-0,013	3,56E-10	4,50E-07	-0,53
RASSF1	cg05989693	3	50 376 155	-0,060	3,02E-07	2,25E-06	1734205	0,006	1,11E-03	4,99E-02	-0,53
PATL2	cg01647795	15	44 969 244	-0,076	8,31E-06	4,14E-05	3229046	0,009	9,23E-08	3,53E-05	-0,53
FAM102A	cg13805537	9	130 728 012	0,090	2,32E-09	3,77E-08	2401779	-0,015	1,33E-10	1,86E-07	-0,53
CHMP7	cg17048073	8	23 102 091	0,096	1,18E-09	2,14E-08	1795933	-0,010	6,94E-09	4,97E-06	-0,53
FBLN2	cg01896761	3	13 590 444	0,106	1,64E-11	6,49E-10	1774602	-0,011	2,16E-19	4,36E-15	-0,53
GNLY	cg16406960	2	85 921 252	-0,066	3,56E-04	1,23E-03	1790692	0,015	2,19E-04	1,66E-02	-0,53
LDLRAP1	cg04344749	1	25 871 814	0,062	4,70E-08	4,65E-07	1809040	-0,008	6,75E-07	1,74E-04	-0,52
ACTN1	cg15867698	14	69 438 267	0,094	1,26E-13	1,97E-11	2232177	-0,017	1,94E-07	6,35E-05	-0,52
PATL2	cg05129597	15	44 970 408	-0,077	3,73E-08	3,84E-07	3229046	0,009	9,23E-08	3,53E-05	-0,52
IFT140	cg09757087	16	1 585 644	0,051	2,63E-03	7,55E-03	1751559	-0,007	1,40E-07	5,07E-05	-0,52
SATB1	cg26768584	3	18 480 242	0,109	2,04E-09	3,39E-08	3247857	-0,007	7,00E-09	4,97E-06	-0,52
PATL2	cg05129597	15	44 970 408	-0,077	3,73E-08	3,84E-07	1768551	0,014	6,76E-09	4,97E-06	-0,52

CRTC3	cg03348466	15	91 104 770	0,058	7,84E-11	2,28E-09	3260715	-0,007	1,09E-04	9,82E-03	-0,52
SPINT2	cg15375239	19	38 755 287	0,065	1,29E-08	1,57E-07	1800739	-0,011	3,68E-08	1,78E-05	-0,52
FAM102A	cg13992008	9	130 739 838	0,103	3,14E-08	3,33E-07	1745112	-0,012	5,49E-09	4,44E-06	-0,52
TTC38	cg04392554	22	46 685 472	-0,057	4,81E-04	1,62E-03	2059886	0,012	9,05E-07	2,27E-04	-0,52
PDGFD	cg04420335	11	103 800 408	-0,075	4,87E-09	7,00E-08	1803094	0,005	4,85E-04	2,90E-02	-0,52
GNLY	cg25066857	2	85 921 438	-0,058	1,50E-03	4,53E-03	1708779	0,012	1,05E-03	4,82E-02	-0,52
KCNT1	cg04927695	9	138 677 436	-0,056	4,91E-10	1,03E-08	1702255	0,004	5,85E-04	3,32E-02	-0,51
CDK5R1	cg07437263	17	30 817 129	0,078	4,04E-09	5,97E-08	1730928	-0,009	2,07E-07	6,71E-05	-0,51
FBLN2	cg01896761	3	13 590 444	0,106	1,64E-11	6,49E-10	2390919	-0,009	1,62E-17	2,18E-13	-0,51
FBLN2	cg18603228	3	13 590 439	0,066	1,19E-09	2,16E-08	2390919	-0,009	1,62E-17	2,18E-13	-0,51
BACH2	cg03035849	6	91 003 200	0,103	3,50E-13	3,87E-11	1670695	-0,007	2,54E-10	3,31E-07	-0,51
PPP2R2B	cg08991927	5	146 436 805	-0,078	3,06E-09	4,73E-08	1660732	0,010	2,02E-04	1,57E-02	-0,51
CD248	cg04702940	11	66 084 599	0,076	3,95E-10	8,62E-09	1726589	-0,012	5,95E-15	4,81E-11	-0,51
S100A10	cg20167074	1	151 967 023	-0,050	2,20E-04	7,93E-04	2046730	0,006	1,66E-04	1,34E-02	-0,51
MCOLN2	cg02621376	1	85 461 303	-0,104	7,38E-06	3,72E-05	1660462	0,011	4,90E-05	5,42E-03	-0,51
C1orf228	cg14658964	1	45 189 026	0,089	3,09E-08	3,28E-07	1663068	-0,014	1,50E-08	8,93E-06	-0,51
S100A10	cg13445177	1	151 967 021	-0,092	1,24E-05	5,92E-05	2046730	0,006	1,66E-04	1,34E-02	-0,51
CD27	cg22036538	12	6 554 051	0,081	2,54E-08	2,78E-07	1688959	-0,014	8,39E-09	5,66E-06	-0,51
KCNT1	cg14435098	9	138 669 066	-0,063	1,04E-05	5,07E-05	1702255	0,004	5,85E-04	3,32E-02	-0,51
GNLY	cg25066857	2	85 921 438	-0,058	1,50E-03	4,53E-03	1790692	0,015	2,19E-04	1,66E-02	-0,50
MAL	cg19762657	2	95 692 524	0,067	2,61E-11	9,38E-10	2320330	-0,015	2,98E-07	9,07E-05	-0,50
SATB1	cg01373248	3	18 480 297	0,065	1,19E-09	2,16E-08	1690646	-0,012	1,61E-10	2,17E-07	-0,50
IFNG	cg01940810	12	68 551 655	-0,084	2,92E-06	1,63E-05	2207291	0,019	3,03E-07	9,16E-05	-0,50
IFT140	cg09757087	16	1 585 644	0,051	2,63E-03	7,55E-03	3241657	-0,007	2,82E-07	8,79E-05	-0,50
CD248	cg07145284	11	66 084 631	0,084	7,84E-08	7,15E-07	1726589	-0,012	5,95E-15	4,81E-11	-0,50
ACTN1	cg26303870	14	69 443 683	0,057	3,24E-10	7,31E-09	2232177	-0,017	1,94E-07	6,35E-05	-0,50
KCNT1	cg13730736	9	138 653 071	-0,054	1,29E-08	1,57E-07	1702255	0,004	5,85E-04	3,32E-02	-0,50
PPP2R2B	cg19494588	5	146 195 103	-0,085	7,88E-07	5,16E-06	1660732	0,010	2,02E-04	1,57E-02	-0,50
TGFBR2	cg04916416	3	30 651 317	0,073	3,24E-05	1,40E-04	2384241	-0,014	2,02E-06	4,37E-04	-0,50
KLHL3	cg13847070	5	137 071 523	0,074	1,28E-06	7,86E-06	1770641	-0,012	3,70E-08	1,78E-05	-0,50
RCAN2	cg19083007	6	46 293 862	-0,110	1,90E-07	1,52E-06	2120210	0,003	6,97E-04	3,74E-02	-0,50
DGKA	cg15354065	12	56 327 217	0,081	7,11E-07	4,72E-06	2319910	-0,011	1,83E-05	2,62E-03	-0,50
RUNX3	cg11336061	1	25 254 149	-0,087	3,66E-05	1,57E-04	1787461	0,005	1,03E-03	4,76E-02	-0,50
CD248	cg13860849	11	66 084 469	0,059	4,62E-07	3,25E-06	1726589	-0,012	5,95E-15	4,81E-11	-0,50
SCRN1	cg08551725	7	30 029 776	0,071	1,09E-08	1,37E-07	1756439	-0,008	3,11E-07	9,34E-05	-0,50
NRCAM	cg09561125	7	108 097 477	0,066	1,89E-07	1,51E-06	2411236	-0,020	2,37E-21	9,60E-17	-0,49
RASSF1	cg20119308	3	50 376 000	-0,079	1,98E-07	1,57E-06	1734205	0,006	1,11E-03	4,99E-02	-0,49
RASSF1	cg05546296	3	50 376 006	-0,103	3,48E-10	7,76E-09	1734205	0,006	1,11E-03	4,99E-02	-0,49
IFT140	cg08006309	16	1 587 810	0,114	5,61E-12	2,83E-10	3241657	-0,007	2,82E-07	8,79E-05	-0,49
RASSF1	cg19811994	3	50 373 641	-0,095	8,13E-09	1,07E-07	1734205	0,006	1,11E-03	4,99E-02	-0,49
RGS10	cg13260278	10	121 265 587	0,117	1,37E-12	1,01E-10	1733538	-0,011	2,26E-11	3,92E-08	-0,49
KCNT1	cg14387909	9	138 681 581	-0,059	6,15E-09	8,49E-08	1702255	0,004	5,85E-04	3,32E-02	-0,49
IFT140	cg01042641	16	1 575 979	0,063	2,72E-08	2,95E-07	1751559	-0,007	1,40E-07	5,07E-05	-0,49

SATB1	cg07108579	3	18 391 124	0,109	9,18E-15	4,38E-12	3247857	-0,007	7,00E-09	4,97E-06	-0,49
SLC22A17	cg10058779	14	23 821 435	0,051	2,23E-10	5,37E-09	1653200	-0,005	1,16E-07	4,27E-05	-0,49
PPP2R2B	cg15021031	5	146 333 034	-0,061	7,96E-06	3,98E-05	2360202	0,004	3,80E-04	2,44E-02	-0,49
NMUR1	cg26913833	2	232 388 288	-0,050	7,60E-05	3,03E-04	1729287	0,002	1,08E-03	4,91E-02	-0,49
FLNB	cg23730027	3	57 995 180	0,075	2,04E-06	1,19E-05	1664922	-0,013	3,39E-12	9,15E-09	-0,49
S100A11	cg10069121	1	152 009 711	-0,066	1,52E-07	1,25E-06	1750101	0,012	5,32E-05	5,73E-03	-0,48
FLT3LG	cg16891376	19	49 976 438	0,060	1,20E-11	5,07E-10	2091412	-0,008	5,44E-04	3,15E-02	-0,48
BACH2	cg19039673	6	90 887 871	0,080	9,85E-10	1,85E-08	1670695	-0,007	2,54E-10	3,31E-07	-0,48
FBLN2	cg18603228	3	13 590 439	0,066	1,19E-09	2,16E-08	1774602	-0,011	2,16E-19	4,36E-15	-0,48
PYHIN1	cg15036529	1	158 900 644	-0,100	2,22E-05	9,99E-05	2395981	0,009	1,56E-06	3,51E-04	-0,48
RCAN2	cg04652496	6	46 294 097	-0,077	4,90E-07	3,42E-06	2120210	0,003	6,97E-04	3,74E-02	-0,48
TTC38	cg00344801	22	46 685 728	-0,056	3,56E-04	1,23E-03	2059886	0,012	9,05E-07	2,27E-04	-0,48
FBLN2	cg12700904	3	13 590 720	0,069	1,25E-11	5,26E-10	1774602	-0,011	2,16E-19	4,36E-15	-0,48
RUNX3	cg05656688	1	25 254 088	-0,062	4,08E-04	1,39E-03	1787461	0,005	1,03E-03	4,76E-02	-0,48
RCAN3	cg11257888	1	24 828 096	0,052	2,53E-04	9,03E-04	1688299	-0,005	6,87E-05	6,82E-03	-0,48
PSMG4	cg20900927	6	3 263 650	0,060	1,24E-10	3,30E-09	3224204	-0,006	3,49E-06	6,74E-04	-0,48
MAL	cg09712683	2	95 692 352	0,082	4,08E-11	1,34E-09	2320330	-0,015	2,98E-07	9,07E-05	-0,48
FAIM3	cg22945467	1	207 096 833	0,061	2,48E-11	9,00E-10	1775542	-0,012	7,00E-08	2,92E-05	-0,48
PTTG1	cg17367077	5	159 850 153	-0,089	2,19E-07	1,72E-06	2042771	0,008	1,75E-07	5,88E-05	-0,48
RASSF1	cg24049629	3	50 376 475	-0,141	1,50E-11	6,07E-10	1734205	0,006	1,11E-03	4,99E-02	-0,48
KLRD1	cg05377120	12	10 456 885	-0,068	2,46E-03	7,10E-03	1799134	0,011	2,14E-04	1,63E-02	-0,48
TGFBR2	cg07613391	3	30 708 746	0,071	1,13E-04	4,35E-04	2384241	-0,014	2,02E-06	4,37E-04	-0,48
RCAN3	cg01768001	1	24 831 147	0,054	1,94E-07	1,54E-06	1688299	-0,005	6,87E-05	6,82E-03	-0,48
IFT140	cg01042641	16	1 575 979	0,063	2,72E-08	2,95E-07	3241657	-0,007	2,82E-07	8,79E-05	-0,47
FBLN2	cg12700904	3	13 590 720	0,069	1,25E-11	5,26E-10	2390919	-0,009	1,62E-17	2,18E-13	-0,47
FAIM3	cg23088126	1	207 078 912	0,091	2,53E-13	3,09E-11	1775542	-0,012	7,00E-08	2,92E-05	-0,47
SERPINE2	cg18681853	2	224 889 133	0,092	6,27E-13	5,75E-11	1655595	-0,007	1,25E-08	7,58E-06	-0,47
GSTM3	cg00297950	1	110 282 525	0,099	9,54E-10	1,80E-08	1736184	-0,005	1,61E-04	1,33E-02	-0,47
DGKA	cg15354065	12	56 327 217	0,081	7,11E-07	4,72E-06	2319913	-0,011	3,48E-05	4,25E-03	-0,47
CDK5R1	cg21855910	17	30 817 397	0,066	1,35E-07	1,13E-06	1730928	-0,009	2,07E-07	6,71E-05	-0,47
MYO15B	cg08781140	17	73 584 617	0,073	2,98E-12	1,77E-10	3248649	-0,009	2,39E-05	3,18E-03	-0,47
LRRN3	cg19798735	7	110 730 805	0,088	9,44E-11	2,66E-09	2048591	-0,009	7,67E-11	1,11E-07	-0,47
IFT140	cg27316811	16	1 576 146	0,077	5,13E-06	2,69E-05	3241657	-0,007	2,82E-07	8,79E-05	-0,47
LTBP3	cg08965235	11	65 325 158	0,078	2,17E-07	1,70E-06	1805395	-0,012	6,47E-09	4,97E-06	-0,47
GSTM3	cg23645476	1	110 282 472	0,080	1,21E-12	9,21E-11	1736184	-0,005	1,61E-04	1,33E-02	-0,47
FBLN2	cg18406197	3	13 590 450	0,063	2,01E-10	4,92E-09	1774602	-0,011	2,16E-19	4,36E-15	-0,47
CDK5R1	cg21855910	17	30 817 397	0,066	1,35E-07	1,13E-06	2062271	-0,005	3,24E-08	1,60E-05	-0,47
SATB1	cg26768584	3	18 480 242	0,109	2,04E-09	3,39E-08	1690646	-0,012	1,61E-10	2,17E-07	-0,47
ACTN1	cg09421468	14	69 446 784	0,060	2,52E-07	1,93E-06	2232177	-0,017	1,94E-07	6,35E-05	-0,47
NUCB2	cg02075820	11	17 300 909	0,098	4,42E-08	4,42E-07	1655913	-0,009	4,40E-13	1,98E-09	-0,47
PTK7	cg07235774	6	43 110 907	0,105	1,10E-13	1,80E-11	2353202	-0,002	8,48E-08	3,30E-05	-0,47
CST7	cg19204859	20	24 933 798	-0,064	8,54E-07	5,53E-06	1679826	0,011	3,22E-05	4,06E-03	-0,47
MCOLN2	cg12277336	1	85 458 682	-0,056	1,21E-05	5,81E-05	1660462	0,011	4,90E-05	5,42E-03	-0,47

KCNT1	cg13473691	9	138 671 650	-0,064	4,43E-08	4,42E-07	1702255	0,004	5,85E-04	3,32E-02	-0,47
FAM102A	cg13992008	9	130 739 838	0,103	3,14E-08	3,33E-07	2401779	-0,015	1,33E-10	1,86E-07	-0,47
RAPGEF1	cg13784312	9	134 609 065	-0,086	2,52E-11	9,10E-10	1769412	0,004	9,23E-04	4,39E-02	-0,46
TGFBR2	cg15171154	3	30 722 557	0,076	6,85E-16	1,12E-12	1726245	-0,013	4,78E-08	2,13E-05	-0,46
TGFBR2	cg04916416	3	30 651 317	0,073	3,24E-05	1,40E-04	1726245	-0,013	4,78E-08	2,13E-05	-0,46
IL15	cg11418177	4	142 636 072	-0,104	4,05E-07	2,90E-06	1724181	0,005	1,99E-06	4,33E-04	-0,46
ZCCHC14	cg16727006	16	87 470 545	0,062	1,29E-03	3,95E-03	1743456	-0,005	1,62E-04	1,33E-02	-0,46
TGFBR2	cg07285675	3	30 667 873	0,053	1,11E-11	4,79E-10	2384241	-0,014	2,02E-06	4,37E-04	-0,46
RUNX3	cg12459932	1	25 292 018	-0,061	1,26E-04	4,79E-04	1787461	0,005	1,03E-03	4,76E-02	-0,46
SCRN1	cg11855325	7	30 029 717	0,089	4,21E-10	9,09E-09	1756439	-0,008	3,11E-07	9,34E-05	-0,46
TGFBR2	cg07285675	3	30 667 873	0,053	1,11E-11	4,79E-10	1726245	-0,013	4,78E-08	2,13E-05	-0,46
TTC38	cg08471335	22	46 685 653	-0,053	2,24E-03	6,52E-03	2059886	0,012	9,05E-07	2,27E-04	-0,46
CDK5R1	cg07437263	17	30 817 129	0,078	4,04E-09	5,97E-08	2062271	-0,005	3,24E-08	1,60E-05	-0,46
CD248	cg13383058	11	66 084 660	0,062	7,78E-10	1,52E-08	1726589	-0,012	5,95E-15	4,81E-11	-0,46
RCAN2	cg06665622	6	46 293 571	-0,067	8,05E-04	2,58E-03	2120210	0,003	6,97E-04	3,74E-02	-0,46
RASSF1	cg01932734	3	50 376 409	-0,080	4,20E-10	9,07E-09	1734205	0,006	1,11E-03	4,99E-02	-0,46
RABGAP1L	cg27299776	1	174 132 239	-0,053	7,83E-06	3,92E-05	1708721	0,005	7,12E-05	6,99E-03	-0,46
PDGFD	cg23852348	11	104 030 857	-0,075	2,83E-07	2,13E-06	1803094	0,005	4,85E-04	2,90E-02	-0,46
SCRN1	cg13366537	7	30 029 754	0,058	3,31E-07	2,44E-06	1756439	-0,008	3,11E-07	9,34E-05	-0,46
RAPGEF1	cg13714407	9	134 604 212	-0,103	2,45E-05	1,09E-04	1769412	0,004	9,23E-04	4,39E-02	-0,45
FAM102A	cg13805537	9	130 728 012	0,090	2,32E-09	3,77E-08	1745112	-0,012	5,49E-09	4,44E-06	-0,45
PPP2R2B	cg08991927	5	146 436 805	-0,078	3,06E-09	4,73E-08	2360202	0,004	3,80E-04	2,44E-02	-0,45
RUNX3	cg23086176	1	25 226 129	-0,081	6,43E-07	4,32E-06	1787461	0,005	1,03E-03	4,76E-02	-0,45
SIK1	cg17610755	21	44 845 927	0,073	7,70E-14	1,45E-11	1717639	-0,016	2,68E-08	1,41E-05	-0,45
GPR56	cg03032770	16	57 669 519	-0,055	1,29E-10	3,41E-09	2384122	0,018	5,05E-06	9,13E-04	-0,45
EPHX2	cg16411256	8	27 348 931	0,061	3,53E-09	5,33E-08	1709237	-0,013	4,60E-11	7,16E-08	-0,45
LTBP3	cg12282552	11	65 321 806	0,076	7,81E-10	1,52E-08	1805395	-0,012	6,47E-09	4,97E-06	-0,45
ROBO3	cg24612667	11	124 735 554	0,053	2,07E-11	7,80E-10	1731561	-0,007	8,36E-05	7,89E-03	-0,45
EFHD2	cg27333269	1	15 739 283	-0,050	2,38E-06	1,36E-05	1761463	0,007	2,95E-04	2,03E-02	-0,45
FGR	cg11587640	1	27 959 253	-0,075	5,99E-09	8,30E-08	1795158	0,015	2,30E-07	7,32E-05	-0,45
GFI1	cg04777348	1	92 952 897	-0,110	6,04E-10	1,23E-08	1690420	0,006	2,39E-04	1,75E-02	-0,44
IFT140	cg27316811	16	1 576 146	0,077	5,13E-06	2,69E-05	1751559	-0,007	1,40E-07	5,07E-05	-0,44
KCNT1	cg02457319	9	138 671 108	-0,077	5,85E-08	5,59E-07	1702255	0,004	5,85E-04	3,32E-02	-0,44
SATB1	cg09086987	3	18 470 432	0,076	5,44E-08	5,26E-07	3247857	-0,007	7,00E-09	4,97E-06	-0,44
PPP2R2B	cg17342759	5	146 461 855	-0,050	1,22E-07	1,04E-06	1660732	0,010	2,02E-04	1,57E-02	-0,44
TGFBR2	cg07613391	3	30 708 746	0,071	1,13E-04	4,35E-04	1726245	-0,013	4,78E-08	2,13E-05	-0,44
LTBP3	cg17969560	11	65 324 768	0,065	3,65E-08	3,77E-07	1805395	-0,012	6,47E-09	4,97E-06	-0,44
IFNG	cg00848007	12	68 553 396	-0,069	1,82E-03	5,40E-03	2207291	0,019	3,03E-07	9,16E-05	-0,44
SCRN1	cg10113101	7	30 029 743	0,071	4,21E-08	4,24E-07	1756439	-0,008	3,11E-07	9,34E-05	-0,44
SPINT2	cg17344048	19	38 756 382	0,080	3,69E-06	2,00E-05	1800739	-0,011	3,68E-08	1,78E-05	-0,44
EPHX2	cg24081819	8	27 348 940	0,054	4,45E-09	6,47E-08	1709237	-0,013	4,60E-11	7,16E-08	-0,44
KCNT1	cg01841415	9	138 679 528	-0,059	7,29E-08	6,72E-07	1702255	0,004	5,85E-04	3,32E-02	-0,44
CST7	cg09971811	20	24 930 099	-0,125	4,90E-12	2,56E-10	1679826	0,011	3,22E-05	4,06E-03	-0,44

LGALS3BP	cg04927537	17	76 976 091	0,070	1,01E-10	2,81E-09	1659688	-0,003	3,33E-05	4,12E-03	-0,44
ESPN	cg00158227	1	6 514 781	0,050	9,37E-10	1,77E-08	1765109	-0,013	8,36E-07	2,11E-04	-0,43
RUNX3	cg15498134	1	25 246 854	-0,074	5,05E-05	2,09E-04	1787461	0,005	1,03E-03	4,76E-02	-0,43
PPFIBP2	cg23039189	11	7 534 028	0,073	1,55E-08	1,84E-07	1675656	-0,008	7,09E-04	3,77E-02	-0,43
CR2	cg10993336	1	207 627 941	0,064	2,45E-10	5,81E-09	2369666	-0,004	6,46E-08	2,78E-05	-0,43
PYHIN1	cg11177526	1	158 900 384	-0,057	5,13E-04	1,71E-03	2395981	0,009	1,56E-06	3,51E-04	-0,43
MAN1C1	cg10555744	1	25 946 258	0,098	5,92E-12	2,95E-10	1713807	-0,007	4,80E-06	8,80E-04	-0,43
RUNX3	cg08544331	1	25 246 922	-0,085	1,24E-05	5,91E-05	1787461	0,005	1,03E-03	4,76E-02	-0,43
ADD2	cg11220663	2	70 994 863	0,051	1,92E-08	2,20E-07	1833858	-0,008	9,29E-10	9,90E-07	-0,43
AKIRIN2	cg05217213	6	88 412 782	-0,066	3,67E-06	2,00E-05	2148847	0,008	6,21E-05	6,43E-03	-0,43
MAL	cg09712683	2	95 692 352	0,082	4,08E-11	1,34E-09	2327860	-0,016	3,82E-08	1,82E-05	-0,43
NT5E	cg23172664	6	86 169 204	0,050	1,23E-10	3,28E-09	1697220	-0,006	1,13E-06	2,73E-04	-0,43
KCNT1	cg13463516	9	138 632 121	-0,067	4,49E-08	4,47E-07	1702255	0,004	5,85E-04	3,32E-02	-0,43
CDH23	cg04179740	10	73 516 760	0,084	2,81E-05	1,23E-04	1783149	-0,010	4,44E-08	2,04E-05	-0,43
PIK3IP1	cg17725019	22	31 677 931	0,083	2,01E-13	2,65E-11	1719986	-0,009	2,21E-06	4,73E-04	-0,43
SIK1	cg17610755	21	44 845 927	0,073	7,70E-14	1,45E-11	3235647	-0,011	2,44E-06	5,03E-04	-0,43
RASSF1	cg00980904	3	50 373 735	-0,077	2,06E-08	2,33E-07	1734205	0,006	1,11E-03	4,99E-02	-0,43
SNX29	cg01366941	16	12 172 962	0,098	2,43E-07	1,87E-06	1659761	-0,004	2,62E-04	1,86E-02	-0,43
GPC2	cg10084644	7	99 775 521	0,080	1,29E-11	5,40E-10	1651642	-0,003	8,65E-04	4,23E-02	-0,43
CD27	cg05900140	12	6 560 162	0,063	4,13E-08	4,17E-07	1688959	-0,014	8,39E-09	5,66E-06	-0,43
SATB1	cg18808261	3	18 464 935	0,089	1,33E-06	8,17E-06	1690646	-0,012	1,61E-10	2,17E-07	-0,42
RUNX3	cg11585280	1	25 292 059	-0,055	2,63E-05	1,16E-04	1787461	0,005	1,03E-03	4,76E-02	-0,42
SATB1	cg07108579	3	18 391 124	0,109	9,18E-15	4,38E-12	1690646	-0,012	1,61E-10	2,17E-07	-0,42
KLRD1	cg05377120	12	10 456 885	-0,068	2,46E-03	7,10E-03	1797988	0,010	2,83E-05	3,65E-03	-0,42
SYTL3	cg12691330	6	159 070 656	-0,100	1,66E-07	1,35E-06	1720623	0,009	2,69E-06	5,47E-04	-0,42
BCL11B	cg14150354	14	99 690 237	0,054	7,01E-07	4,67E-06	1665761	-0,010	1,07E-03	4,89E-02	-0,42
GPC2	cg04778012	7	99 775 508	0,071	6,52E-09	8,91E-08	1651642	-0,003	8,65E-04	4,23E-02	-0,42
TCF7	cg18338046	5	133 452 188	0,102	2,02E-08	2,29E-07	1676470	-0,005	8,18E-08	3,22E-05	-0,42
PLCG1	cg24961795	20	39 769 764	0,055	1,35E-03	4,13E-03	2382906	-0,006	8,02E-04	4,01E-02	-0,42
NRCAM	cg06570167	7	108 095 719	0,057	1,09E-09	2,01E-08	1722809	-0,008	3,07E-16	3,11E-12	-0,42
TCF7	cg20682563	5	133 460 874	0,075	3,72E-08	3,83E-07	1676470	-0,005	8,18E-08	3,22E-05	-0,42
CHD7	cg27519392	8	61 778 137	0,058	9,78E-07	6,23E-06	1677376	-0,006	8,96E-05	8,34E-03	-0,42
TCF7	cg25947408	5	133 451 383	0,058	3,40E-05	1,46E-04	1676470	-0,005	8,18E-08	3,22E-05	-0,42
RNF144A	cg20437098	2	7 059 760	0,072	2,74E-14	7,68E-12	1900263	-0,011	5,32E-06	9,40E-04	-0,42
TCF7	cg02387618	5	133 453 480	0,121	5,80E-12	2,91E-10	1676470	-0,005	8,18E-08	3,22E-05	-0,42
SOX13	cg22346959	1	204 082 391	-0,066	3,72E-11	1,24E-09	1683755	0,006	3,30E-05	4,11E-03	-0,42
LRRN3	cg06545367	7	110 731 527	0,078	2,50E-08	2,74E-07	2048591	-0,009	7,67E-11	1,11E-07	-0,42
EDARADD	cg18964582	1	236 557 182	-0,052	6,59E-09	8,99E-08	1761820	0,009	1,79E-05	2,58E-03	-0,42
SYTL3	cg11065271	6	159 070 607	-0,111	4,42E-11	1,43E-09	1720623	0,009	2,69E-06	5,47E-04	-0,41
SATB1	cg19818826	3	18 467 816	0,088	1,95E-06	1,14E-05	1690646	-0,012	1,61E-10	2,17E-07	-0,41
SNX29	cg00459119	16	12 172 834	0,086	9,41E-11	2,65E-09	1659761	-0,004	2,62E-04	1,86E-02	-0,41
SLC22A17	cg23464698	14	23 822 265	0,059	4,25E-09	6,23E-08	1653200	-0,005	1,16E-07	4,27E-05	-0,41
MMP28	cg09807148	17	34 122 069	0,051	7,31E-09	9,81E-08	2399016	-0,007	1,02E-12	3,74E-09	-0,41

NKG7	cg10126923	19	51 875 451	-0,090	8,45E-09	1,11E-07	1682993	0,012	7,53E-05	7,23E-03	-0,41
RUNX3	cg16529592	1	25 292 215	-0,061	3,16E-05	1,37E-04	1787461	0,005	1,03E-03	4,76E-02	-0,41
NDRG1	cg08691775	8	134 310 658	-0,057	5,40E-04	1,80E-03	1809931	0,006	4,49E-05	5,08E-03	-0,41
CDH23	cg23282441	10	73 533 927	0,061	2,20E-05	9,91E-05	1783149	-0,010	4,44E-08	2,04E-05	-0,41
FBLN2	cg18406197	3	13 590 450	0,063	2,01E-10	4,92E-09	2390919	-0,009	1,62E-17	2,18E-13	-0,41
GPC2	cg15140703	7	99 775 532	0,093	5,28E-10	1,10E-08	1651642	-0,003	8,65E-04	4,23E-02	-0,41
ROBO3	cg09251429	11	124 735 128	0,058	1,82E-11	7,05E-10	1731561	-0,007	8,36E-05	7,89E-03	-0,41
TRIM69	cg05439368	15	45 028 098	-0,106	6,84E-12	3,29E-10	1813430	0,003	7,89E-05	7,53E-03	-0,41
MAL	cg10108468	2	95 719 390	0,059	5,53E-10	1,14E-08	2320330	-0,015	2,98E-07	9,07E-05	-0,41
ZNF365	cg14701867	10	64 193 068	-0,055	1,62E-07	1,32E-06	1658094	0,004	7,38E-05	7,17E-03	-0,41
MCOLN2	cg01413809	1	85 462 483	-0,066	4,05E-12	2,22E-10	1660462	0,011	4,90E-05	5,42E-03	-0,41
DCHS1	cg09236382	11	6 676 366	0,073	1,33E-10	3,50E-09	1739640	-0,003	2,57E-08	1,38E-05	-0,41
TGFBR2	cg15171154	3	30 722 557	0,076	6,85E-16	1,12E-12	2384241	-0,014	2,02E-06	4,37E-04	-0,41
MAL	cg19762657	2	95 692 524	0,067	2,61E-11	9,38E-10	2327860	-0,016	3,82E-08	1,82E-05	-0,41
ROBO1	cg07997035	3	79 816 949	0,051	1,53E-09	2,67E-08	1806790	-0,003	4,96E-07	1,40E-04	-0,41
NKG7	cg04264075	19	51 876 470	-0,084	5,10E-10	1,06E-08	1682993	0,012	7,53E-05	7,23E-03	-0,40
GPC2	cg18691434	7	99 775 425	0,079	7,68E-14	1,44E-11	1651642	-0,003	8,65E-04	4,23E-02	-0,40
ROBO1	cg15709235	3	79 068 292	0,089	5,39E-15	3,20E-12	1806790	-0,003	4,96E-07	1,40E-04	-0,40
IGF1R	cg01631855	15	99 194 172	0,074	6,99E-13	6,22E-11	1744023	-0,008	1,21E-09	1,19E-06	-0,40
SATB1	cg09086987	3	18 470 432	0,076	5,44E-08	5,26E-07	1690646	-0,012	1,61E-10	2,17E-07	-0,40
PPFIBP2	cg01842720	11	7 535 887	0,052	1,29E-06	7,93E-06	1675656	-0,008	7,09E-04	3,77E-02	-0,40
PDE9A	cg08729135	21	44 073 555	0,052	5,44E-08	5,26E-07	1683063	-0,004	3,02E-06	6,05E-04	-0,40
KLHL29	cg11227278	2	23 749 277	0,076	7,68E-08	7,02E-07	3178406	-0,004	2,58E-05	3,36E-03	-0,40
PPP2R2B	cg17342759	5	146 461 855	-0,050	1,22E-07	1,04E-06	2360202	0,004	3,80E-04	2,44E-02	-0,40
TRAF3IP2	cg15931839	6	111 880 530	0,091	6,84E-16	1,12E-12	1701514	-0,007	1,50E-05	2,21E-03	-0,40
ROBO3	cg15221604	11	124 738 735	0,112	1,07E-12	8,38E-11	1731561	-0,007	8,36E-05	7,89E-03	-0,40
ESPN	cg03397716	1	6 484 980	0,068	1,40E-12	1,02E-10	1765109	-0,013	8,36E-07	2,11E-04	-0,40
MYO15B	cg06390536	17	73 584 068	0,063	1,32E-11	5,47E-10	3248649	-0,009	2,39E-05	3,18E-03	-0,40
ESPN	cg12406391	1	6 520 194	0,091	3,27E-09	5,01E-08	1765109	-0,013	8,36E-07	2,11E-04	-0,40
SCRN1	cg06133110	7	30 028 307	0,070	8,36E-06	4,16E-05	1756439	-0,008	3,11E-07	9,34E-05	-0,40
PTTG1	cg26775866	5	159 849 193	-0,150	2,93E-16	6,94E-13	2042771	0,008	1,75E-07	5,88E-05	-0,39
SCRN1	cg10589443	7	30 028 281	0,059	4,01E-05	1,70E-04	1756439	-0,008	3,11E-07	9,34E-05	-0,39
ESPN	cg26815404	1	6 500 700	0,064	2,13E-10	5,17E-09	1765109	-0,013	8,36E-07	2,11E-04	-0,39
MATK	cg13387994	19	3 786 957	-0,061	1,89E-07	1,51E-06	2319000	0,009	2,00E-04	1,56E-02	-0,39
CLDND2	cg12011479	19	51 873 045	-0,056	2,18E-05	9,82E-05	2077680	0,013	3,62E-07	1,07E-04	-0,39
SATB1	cg21902966	3	18 391 029	0,104	9,48E-15	4,42E-12	3247857	-0,007	7,00E-09	4,97E-06	-0,39
PPP2R2B	cg19494588	5	146 195 103	-0,085	7,88E-07	5,16E-06	2360202	0,004	3,80E-04	2,44E-02	-0,39
SPEG	cg02323356	2	220 313 153	0,074	3,02E-14	8,15E-12	1740052	-0,003	6,40E-05	6,51E-03	-0,39
ENTPD3	cg10755058	3	40 428 713	0,083	2,71E-10	6,30E-09	1775114	-0,002	3,76E-04	2,42E-02	-0,39
FAM179A	cg05707236	2	29 204 695	-0,062	6,27E-08	5,93E-07	3247182	0,008	3,19E-04	2,15E-02	-0,39
MAL	cg03129884	2	95 692 727	0,065	1,23E-10	3,29E-09	2320330	-0,015	2,98E-07	9,07E-05	-0,39
ACTN1	cg08170227	14	69 422 517	0,055	2,43E-09	3,92E-08	2232177	-0,017	1,94E-07	6,35E-05	-0,39
TGFBR2	cg13724812	3	30 689 835	0,106	1,02E-10	2,83E-09	1726245	-0,013	4,78E-08	2,13E-05	-0,39

ROBO1	cg19258784	3	79 067 596	0,081	8,60E-14	1,54E-11	1806790	-0,003	4,96E-07	1,40E-04	-0,39
FAM134B	cg00461022	5	16 618 052	0,115	1,18E-13	1,88E-11	1811330	-0,004	3,52E-06	6,76E-04	-0,39
DTHD1	cg00682487	4	36 283 275	-0,073	3,97E-04	1,36E-03	1765773	0,007	1,43E-06	3,31E-04	-0,38
FGR	cg11587640	1	27 959 253	-0,075	5,99E-09	8,30E-08	2368318	0,017	8,84E-08	3,41E-05	-0,38
PDGFRB	cg26247309	5	149 501 835	-0,079	5,11E-11	1,61E-09	1815057	0,006	9,48E-05	8,74E-03	-0,38
ESPN	cg17430636	1	6 500 410	0,053	7,92E-11	2,30E-09	1765109	-0,013	8,36E-07	2,11E-04	-0,38
OSBPL5	cg06131936	11	3 187 271	-0,100	2,87E-10	6,61E-09	1802151	0,008	8,45E-04	4,16E-02	-0,38
ZNF263	cg05622324	16	3 334 135	0,067	4,04E-08	4,10E-07	1692620	-0,005	4,13E-04	2,57E-02	-0,38
RAPGEF1	cg07285276	9	134 613 015	-0,072	4,67E-08	4,62E-07	1769412	0,004	9,23E-04	4,39E-02	-0,38
BCL11B	cg23479730	14	99 681 757	0,088	3,91E-10	8,55E-09	1665761	-0,010	1,07E-03	4,89E-02	-0,38
OSBPL5	cg11219178	11	3 120 949	-0,051	1,44E-06	8,72E-06	2307032	0,009	2,71E-05	3,52E-03	-0,38
ALOX5AP	cg02982610	13	31 309 403	-0,069	5,50E-07	3,77E-06	1797875	0,008	7,96E-04	4,00E-02	-0,38
NRCAM	cg09561125	7	108 097 477	0,066	1,89E-07	1,51E-06	1722809	-0,008	3,07E-16	3,11E-12	-0,38
FLNB	cg12194336	3	58 067 906	0,082	8,19E-10	1,58E-08	1664922	-0,013	3,39E-12	9,15E-09	-0,38
CR2	cg04439623	1	207 627 601	0,055	2,37E-08	2,62E-07	2369666	-0,004	6,46E-08	2,78E-05	-0,38
DIP2B	cg05512157	12	50 901 878	0,085	6,89E-08	6,42E-07	2180352	-0,009	6,12E-05	6,40E-03	-0,38
IGF1R	cg03795574	15	99 256 874	0,062	1,24E-08	1,52E-07	1744023	-0,008	1,21E-09	1,19E-06	-0,38
ROBO3	cg10680051	11	124 735 089	0,086	3,95E-12	2,18E-10	1731561	-0,007	8,36E-05	7,89E-03	-0,38
GFI1	cg04955573	1	92 944 306	-0,092	1,55E-08	1,84E-07	1690420	0,006	2,39E-04	1,75E-02	-0,38
IFNG	cg26227465	12	68 553 577	-0,087	2,40E-04	8,59E-04	2207291	0,019	3,03E-07	9,16E-05	-0,38
MMP28	cg12700449	17	34 121 837	0,073	8,55E-10	1,64E-08	2399016	-0,007	1,02E-12	3,74E-09	-0,38
TRAF3IP2	cg15772366	6	111 926 749	0,109	2,64E-07	2,01E-06	1701514	-0,007	1,50E-05	2,21E-03	-0,38
FAM179A	cg16944977	2	29 203 230	-0,068	6,02E-08	5,73E-07	3247182	0,008	3,19E-04	2,15E-02	-0,37
SPEG	cg12213062	2	220 299 653	0,087	6,88E-11	2,05E-09	1740052	-0,003	6,40E-05	6,51E-03	-0,37
ZCCHC14	cg10375267	16	87 523 700	0,100	4,32E-12	2,33E-10	1743456	-0,005	1,62E-04	1,33E-02	-0,37
DEAF1	cg21156386	11	692 983	0,093	3,16E-11	1,09E-09	1689868	-0,003	7,11E-04	3,78E-02	-0,37
GPC2	cg13210467	7	99 775 443	0,074	7,49E-14	1,42E-11	1651642	-0,003	8,65E-04	4,23E-02	-0,37
DGKA	cg10782923	12	56 329 731	0,092	2,76E-10	6,40E-09	2319910	-0,011	1,83E-05	2,62E-03	-0,37
MAN1C1	cg09027133	1	25 945 042	0,055	8,27E-08	7,47E-07	1713807	-0,007	4,80E-06	8,80E-04	-0,37
MAL	cg03129884	2	95 692 727	0,065	1,23E-10	3,29E-09	2327860	-0,016	3,82E-08	1,82E-05	-0,37
BCL11B	cg02963266	14	99 681 710	0,136	4,95E-14	1,10E-11	1665761	-0,010	1,07E-03	4,89E-02	-0,37
IGF1R	cg13297560	15	99 320 054	0,095	5,97E-12	2,97E-10	1744023	-0,008	1,21E-09	1,19E-06	-0,37
DHRS3	cg13314614	1	12 655 992	0,054	3,50E-08	3,64E-07	1752478	-0,007	5,49E-04	3,17E-02	-0,37
ID3	cg25287482	1	23 884 847	0,067	1,20E-09	2,18E-08	1732296	-0,008	2,48E-06	5,07E-04	-0,37
IGF1R	cg08729686	15	99 194 624	0,087	1,27E-10	3,36E-09	1744023	-0,008	1,21E-09	1,19E-06	-0,37
TCF7	cg15413523	5	133 451 625	0,057	6,83E-07	4,55E-06	1676470	-0,005	8,18E-08	3,22E-05	-0,37
TRAF3IP2	cg21699035	6	111 926 911	0,069	1,72E-07	1,39E-06	1701514	-0,007	1,50E-05	2,21E-03	-0,37
PDGFRB	cg14051336	5	149 515 317	-0,054	1,39E-08	1,68E-07	1815057	0,006	9,48E-05	8,74E-03	-0,37
ACTN1	cg00739471	14	69 415 588	0,122	3,30E-15	2,44E-12	2232177	-0,017	1,94E-07	6,35E-05	-0,37
RABGAP1L	cg07476963	1	174 127 462	-0,078	2,97E-09	4,62E-08	1708721	0,005	7,12E-05	6,99E-03	-0,37
GFI1	cg25320328	1	92 953 037	-0,124	5,36E-11	1,67E-09	1690420	0,006	2,39E-04	1,75E-02	-0,37
ROBO1	cg24784794	3	79 068 831	0,060	2,37E-09	3,83E-08	1806790	-0,003	4,96E-07	1,40E-04	-0,37
DGKA	cg10782923	12	56 329 731	0,092	2,76E-10	6,40E-09	2319913	-0,011	3,48E-05	4,25E-03	-0,37

DTHD1	cg21010701	4	36 283 195	-0,065	6,80E-04	2,21E-03	1765773	0,007	1,43E-06	3,31E-04	-0,37
ARHGEF4	cg23415434	2	131 798 869	0,061	9,60E-09	1,23E-07	1666076	-0,002	1,85E-05	2,63E-03	-0,36
GSTP1	cg22224704	11	67 352 041	-0,061	4,49E-09	6,52E-08	1679809	0,006	1,67E-04	1,34E-02	-0,36
MAN1C1	cg16836311	1	25 944 712	0,091	6,33E-08	5,98E-07	1713807	-0,007	4,80E-06	8,80E-04	-0,36
RCAN3	cg15519096	1	24 833 311	0,081	3,00E-04	1,05E-03	1688299	-0,005	6,87E-05	6,82E-03	-0,36
LGALS3BP	cg25178683	17	76 976 267	0,062	7,36E-11	2,16E-09	1659688	-0,003	3,33E-05	4,12E-03	-0,36
BZW2	cg03976645	7	16 724 981	0,082	7,17E-10	1,42E-08	1676548	-0,011	2,82E-08	1,46E-05	-0,36
FBXO15	cg25740500	18	71 749 983	0,051	3,14E-11	1,09E-09	1755281	-0,003	5,89E-08	2,57E-05	-0,36
CDK5R1	cg19634527	17	30 814 814	0,095	3,81E-08	3,91E-07	2062271	-0,005	3,24E-08	1,60E-05	-0,36
BCL11B	cg10178917	14	99 665 210	0,070	4,46E-12	2,39E-10	1665761	-0,010	1,07E-03	4,89E-02	-0,36
TRIM69	cg22107533	15	45 028 083	-0,087	6,66E-12	3,22E-10	1813430	0,003	7,89E-05	7,53E-03	-0,36
FAM179A	cg23167594	2	29 204 200	-0,059	3,42E-07	2,51E-06	3247182	0,008	3,19E-04	2,15E-02	-0,36
GPC2	cg00553149	7	99 775 558	0,062	1,34E-07	1,13E-06	1651642	-0,003	8,65E-04	4,23E-02	-0,36
LILRB1	cg05365532	19	55 128 350	-0,106	4,33E-09	6,33E-08	1708248	0,002	2,52E-04	1,80E-02	-0,36
ZNF256	cg13359948	19	58 458 769	0,056	1,14E-09	2,08E-08	1757408	-0,009	5,63E-07	1,54E-04	-0,36
IFT140	cg07013955	16	1 595 532	0,084	6,91E-07	4,60E-06	1751559	-0,007	1,40E-07	5,07E-05	-0,36
PTTG1	cg23024444	5	159 848 299	-0,067	1,39E-06	8,48E-06	2042771	0,008	1,75E-07	5,88E-05	-0,36
GAS7	cg26999423	17	10 017 891	-0,059	5,06E-08	4,94E-07	1745994	0,007	3,68E-04	2,38E-02	-0,36
DGKA	cg25416125	12	56 329 615	0,085	3,71E-10	8,18E-09	2319913	-0,011	3,48E-05	4,25E-03	-0,36
SATB1	cg21902966	3	18 391 029	0,104	9,48E-15	4,42E-12	1690646	-0,012	1,61E-10	2,17E-07	-0,36
SREBF1	cg08129017	17	17 728 660	0,067	8,79E-06	4,35E-05	1663035	-0,010	4,98E-07	1,40E-04	-0,36
GFI1	cg24517501	1	92 952 702	-0,104	6,50E-11	1,96E-09	1690420	0,006	2,39E-04	1,75E-02	-0,36
OPRM1	cg03081675	6	154 540 097	0,099	2,04E-10	4,99E-09	1796497	-0,005	6,53E-04	3,58E-02	-0,36
RASSF4	cg11753018	10	45 469 396	-0,089	8,05E-10	1,56E-08	1690566	0,004	2,98E-05	3,81E-03	-0,36
SLC22A17	cg17199325	14	23 821 229	0,052	3,44E-06	1,89E-05	1653200	-0,005	1,16E-07	4,27E-05	-0,36
SLC4A4	cg00650386	4	72 248 830	-0,063	6,35E-09	8,72E-08	1734897	0,003	2,22E-04	1,68E-02	-0,36
PLEK	cg13468685	2	68 592 737	-0,109	5,41E-11	1,68E-09	1795762	0,013	1,19E-04	1,05E-02	-0,35
S100A10	cg26230275	1	151 965 283	-0,067	5,54E-09	7,77E-08	2046730	0,006	1,66E-04	1,34E-02	-0,35
HOOK1	cg21577889	1	60 281 077	0,059	9,58E-11	2,69E-09	1741406	-0,004	4,11E-04	2,56E-02	-0,35
CLDND2	cg14496375	19	51 872 365	-0,075	1,98E-04	7,22E-04	2077680	0,013	3,62E-07	1,07E-04	-0,35
SNX29	cg00459119	16	12 172 834	0,086	9,41E-11	2,65E-09	1670539	-0,007	4,15E-04	2,58E-02	-0,35
ROBO1	cg08661007	3	79 817 232	0,075	1,59E-10	4,05E-09	1806790	-0,003	4,96E-07	1,40E-04	-0,35
IFNG	cg05224770	12	68 553 819	-0,056	8,13E-03	2,07E-02	2207291	0,019	3,03E-07	9,16E-05	-0,35
IGF1R	cg07779120	15	99 194 021	0,065	5,53E-10	1,14E-08	1744023	-0,008	1,21E-09	1,19E-06	-0,35
MAN1C1	cg04116354	1	26 003 643	0,070	1,67E-09	2,87E-08	1713807	-0,007	4,80E-06	8,80E-04	-0,35
MAL	cg10108468	2	95 719 390	0,059	5,53E-10	1,14E-08	2327860	-0,016	3,82E-08	1,82E-05	-0,35
RGS10	cg05241536	10	121 298 236	0,077	1,49E-09	2,60E-08	1733538	-0,011	2,26E-11	3,92E-08	-0,35
TBC1D4	cg21949157	13	76 054 909	0,067	4,43E-09	6,46E-08	1688098	-0,010	1,95E-07	6,35E-05	-0,35
DOK2	cg08288130	8	21 771 540	-0,067	2,02E-10	4,95E-09	1791211	0,008	2,27E-04	1,70E-02	-0,35
OSBPL5	cg11219178	11	3 120 949	-0,051	1,44E-06	8,72E-06	1802151	0,008	8,45E-04	4,16E-02	-0,35
GDPD5	cg01522592	11	75 235 311	-0,055	8,47E-06	4,21E-05	1701643	0,010	1,54E-04	1,28E-02	-0,35
NT5E	cg27039625	6	86 159 096	0,094	2,71E-13	3,24E-11	1697220	-0,006	1,13E-06	2,73E-04	-0,35
SPEG	cg15634877	2	220 299 643	0,071	2,64E-10	6,17E-09	1740052	-0,003	6,40E-05	6,51E-03	-0,35

KCNT1	cg14341131	9	138 605 049	-0,058	9,87E-07	6,27E-06	1702255	0,004	5,85E-04	3,32E-02	-0,35
PDGFD	cg04420335	11	103 800 408	-0,075	4,87E-09	7,00E-08	2376859	0,003	5,46E-04	3,16E-02	-0,35
CDH23	cg08293528	10	73 565 688	0,053	3,71E-09	5,56E-08	1783149	-0,010	4,44E-08	2,04E-05	-0,34
ALDH3B1	cg05620821	11	67 777 618	-0,058	1,71E-08	2,00E-07	1728662	0,003	9,38E-04	4,44E-02	-0,34
CNST	cg11812748	1	246 733 057	0,067	1,12E-08	1,40E-07	1670263	-0,008	6,17E-05	6,42E-03	-0,34
APBA2	cg21917349	15	29 213 860	0,058	1,32E-03	4,04E-03	1723626	-0,010	4,50E-10	5,35E-07	-0,34
SNX29	cg01366941	16	12 172 962	0,098	2,43E-07	1,87E-06	1670539	-0,007	4,15E-04	2,58E-02	-0,34
DGKA	cg07679948	12	56 329 641	0,115	2,33E-10	5,57E-09	2319913	-0,011	3,48E-05	4,25E-03	-0,34
KLHL29	cg21206147	2	23 749 087	0,072	1,15E-05	5,55E-05	3178406	-0,004	2,58E-05	3,36E-03	-0,34
ROBO3	cg08522176	11	124 735 398	0,057	4,35E-10	9,35E-09	1731561	-0,007	8,36E-05	7,89E-03	-0,34
SLC4A4	cg19850370	4	72 205 104	-0,059	9,32E-06	4,58E-05	1734897	0,003	2,22E-04	1,68E-02	-0,34
TMEM87A	cg23983173	15	42 564 634	-0,058	2,91E-08	3,12E-07	1716816	0,004	8,44E-04	4,16E-02	-0,34
ROBO1	cg04562217	3	79 815 718	0,084	7,41E-11	2,18E-09	1806790	-0,003	4,96E-07	1,40E-04	-0,34
ROBO1	cg11980129	3	79 815 639	0,071	1,49E-09	2,61E-08	1806790	-0,003	4,96E-07	1,40E-04	-0,34
PTK7	cg25033767	6	43 044 876	0,058	1,55E-09	2,69E-08	2353202	-0,002	8,48E-08	3,30E-05	-0,34
FBLN2	cg25120326	3	13 591 084	0,058	2,72E-07	2,06E-06	1774602	-0,011	2,16E-19	4,36E-15	-0,34
CITED4	cg13150171	1	41 326 892	0,067	4,44E-10	9,50E-09	1787691	-0,007	2,59E-08	1,38E-05	-0,34
DGKA	cg07679948	12	56 329 641	0,115	2,33E-10	5,57E-09	2319910	-0,011	1,83E-05	2,62E-03	-0,34
PTK7	cg21663580	6	43 044 771	0,065	3,65E-08	3,77E-07	2353202	-0,002	8,48E-08	3,30E-05	-0,34
GPC2	cg00048759	7	99 775 422	0,066	3,60E-14	8,99E-12	1651642	-0,003	8,65E-04	4,23E-02	-0,34
DGKA	cg06915826	12	56 329 903	0,112	1,24E-11	5,23E-10	2319913	-0,011	3,48E-05	4,25E-03	-0,34
DHRS3	cg06641388	1	12 655 240	0,051	3,76E-12	2,10E-10	1752478	-0,007	5,49E-04	3,17E-02	-0,34
TMIGD2	cg11980819	19	4 302 579	0,058	3,31E-08	3,48E-07	2160577	-0,004	8,06E-07	2,06E-04	-0,34
DGKA	cg06915826	12	56 329 903	0,112	1,24E-11	5,23E-10	2319910	-0,011	1,83E-05	2,62E-03	-0,34
ITGA6	cg10155853	2	173 293 328	0,069	3,22E-10	7,27E-09	2359287	-0,009	2,67E-07	8,37E-05	-0,34
ALDH5A1	cg25375764	6	24 494 665	0,051	2,93E-08	3,14E-07	2372398	-0,004	6,13E-06	1,06E-03	-0,34
ESPN	cg26419880	1	6 515 624	0,074	7,46E-12	3,52E-10	1765109	-0,013	8,36E-07	2,11E-04	-0,34
SPEG	cg02487130	2	220 349 733	0,069	1,53E-12	1,09E-10	1740052	-0,003	6,40E-05	6,51E-03	-0,34
DGKA	cg25416125	12	56 329 615	0,085	3,71E-10	8,18E-09	2319910	-0,011	1,83E-05	2,62E-03	-0,34
GFI1	cg23624705	1	92 952 655	-0,118	6,64E-12	3,22E-10	1690420	0,006	2,39E-04	1,75E-02	-0,34
RCAN3	cg17854320	1	24 829 245	0,070	1,50E-12	1,07E-10	1688299	-0,005	6,87E-05	6,82E-03	-0,34
CRLF1	cg13407975	19	18 705 946	0,099	1,32E-10	3,47E-09	2368576	-0,007	1,87E-05	2,64E-03	-0,34
LRRC16A	cg21668832	6	25 278 956	0,057	4,69E-06	2,48E-05	3244461	-0,002	2,33E-04	1,72E-02	-0,33
ID3	cg02343604	1	23 884 703	0,093	1,94E-13	2,59E-11	1732296	-0,008	2,48E-06	5,07E-04	-0,33
SERINC5	cg02417427	5	79 479 211	0,060	4,56E-05	1,91E-04	1789464	-0,008	1,20E-07	4,38E-05	-0,33
GFI1	cg20125091	1	92 952 641	-0,051	2,77E-07	2,10E-06	1690420	0,006	2,39E-04	1,75E-02	-0,33
PDGFD	cg23852348	11	104 030 857	-0,075	2,83E-07	2,13E-06	2376859	0,003	5,46E-04	3,16E-02	-0,33
EDARADD	cg09809672	1	236 557 682	-0,076	1,82E-08	2,10E-07	1761820	0,009	1,79E-05	2,58E-03	-0,33
ZNF75A	cg07949597	16	3 355 079	0,056	3,05E-08	3,24E-07	3186390	-0,006	5,90E-04	3,34E-02	-0,33
HOOK1	cg23996071	1	60 280 809	0,069	2,30E-10	5,51E-09	1741406	-0,004	4,11E-04	2,56E-02	-0,33
LIMK2	cg01606027	22	31 607 212	-0,058	2,82E-05	1,24E-04	2367671	0,001	5,87E-04	3,32E-02	-0,33
GAS7	cg22541400	17	9 840 728	-0,059	4,59E-08	4,56E-07	1745994	0,007	3,68E-04	2,38E-02	-0,33
LTK	cg25298161	15	41 806 135	0,058	4,99E-11	1,58E-09	1679290	-0,005	4,55E-05	5,11E-03	-0,32

KLHL29	cg15123692	2	23 886 357	0,072	3,37E-12	1,94E-10	3178406	-0,004	2,58E-05	3,36E-03	-0,32
LILRB1	cg01720520	19	55 127 892	-0,060	1,49E-07	1,23E-06	1708248	0,002	2,52E-04	1,80E-02	-0,32
MAN1C1	cg16585234	1	25 944 802	0,072	2,94E-07	2,20E-06	1713807	-0,007	4,80E-06	8,80E-04	-0,32
DOK2	cg15316716	8	21 769 797	-0,060	6,76E-04	2,20E-03	1791211	0,008	2,27E-04	1,70E-02	-0,32
OXNAD1	cg22230912	3	16 331 335	0,072	1,27E-06	7,81E-06	3244030	-0,003	2,89E-04	2,00E-02	-0,32
ITGA6	cg19134705	2	173 293 182	0,062	5,14E-09	7,31E-08	2359287	-0,009	2,67E-07	8,37E-05	-0,32
LTK	cg07621749	15	41 806 141	0,053	2,50E-10	5,91E-09	1679290	-0,005	4,55E-05	5,11E-03	-0,32
ESPN	cg10210806	1	6 514 605	0,055	2,35E-06	1,35E-05	1765109	-0,013	8,36E-07	2,11E-04	-0,32
ADAP1	cg18664596	7	947 151	-0,052	1,10E-06	6,88E-06	2047511	0,010	9,10E-04	4,35E-02	-0,32
RCAN3	cg00917251	1	24 829 987	0,056	1,30E-05	6,16E-05	1688299	-0,005	6,87E-05	6,82E-03	-0,32
TRIM69	cg15022400	15	45 028 161	-0,062	2,56E-09	4,09E-08	1813430	0,003	7,89E-05	7,53E-03	-0,32
ATP2B4	cg23598089	1	203 652 079	-0,050	1,56E-05	7,26E-05	1680579	0,008	1,15E-05	1,81E-03	-0,32
ROBO3	cg12859211	11	124 735 105	0,064	5,72E-10	1,17E-08	1731561	-0,007	8,36E-05	7,89E-03	-0,32
CNN3	cg14669167	1	95 391 585	0,060	9,90E-10	1,85E-08	1782439	-0,003	9,73E-08	3,68E-05	-0,32
SREBF1	cg08129017	17	17 728 660	0,067	8,79E-06	4,35E-05	2328986	-0,006	7,31E-05	7,15E-03	-0,32
HOOK1	cg15900248	1	60 280 812	0,093	2,52E-09	4,03E-08	1741406	-0,004	4,11E-04	2,56E-02	-0,32
NLGN2	cg15660077	17	7 311 620	0,055	3,64E-07	2,65E-06	1764158	-0,004	9,47E-06	1,55E-03	-0,32
ASGR2	cg26661623	17	7 019 262	-0,081	3,04E-11	1,06E-09	1694966	0,003	9,53E-04	4,51E-02	-0,32
CLEC11A	cg01130127	19	51 228 821	0,080	5,36E-12	2,74E-10	1807359	-0,004	6,14E-04	3,42E-02	-0,32
SATB1	cg19818826	3	18 467 816	0,088	1,95E-06	1,14E-05	3247857	-0,007	7,00E-09	4,97E-06	-0,32
CNN3	cg26619317	1	95 392 389	0,086	2,79E-13	3,30E-11	1782439	-0,003	9,73E-08	3,68E-05	-0,32
RNF144A	cg20437098	2	7 059 760	0,072	2,74E-14	7,68E-12	1777660	-0,007	4,67E-05	5,23E-03	-0,31
SOX12	cg03901958	20	308 226	0,067	1,06E-10	2,91E-09	1736974	-0,002	1,47E-05	2,20E-03	-0,31
APBA2	cg17716765	15	29 407 853	0,093	7,23E-12	3,43E-10	1723626	-0,010	4,50E-10	5,35E-07	-0,31
OSBPL5	cg06131936	11	3 187 271	-0,100	2,87E-10	6,61E-09	2307032	0,009	2,71E-05	3,52E-03	-0,31
TRAF3IP2	cg26379258	6	111 888 446	0,090	9,38E-09	1,21E-07	1701514	-0,007	1,50E-05	2,21E-03	-0,31
ROBO3	cg17272642	11	124 747 244	0,060	1,87E-09	3,15E-08	1731561	-0,007	8,36E-05	7,89E-03	-0,31
TMIGD2	cg03757250	19	4 302 846	0,069	9,77E-13	7,86E-11	2160577	-0,004	8,06E-07	2,06E-04	-0,31
CLCF1	cg14588642	11	67 139 423	-0,074	8,73E-10	1,67E-08	1661197	0,005	7,40E-04	3,85E-02	-0,31
CNN3	cg18344652	1	95 393 096	0,098	6,58E-11	1,98E-09	1782439	-0,003	9,73E-08	3,68E-05	-0,31
SPEG	cg16440561	2	220 312 854	0,149	2,23E-10	5,37E-09	1740052	-0,003	6,40E-05	6,51E-03	-0,31
BLK	cg16861076	8	11 421 594	0,052	3,89E-08	3,97E-07	1668277	-0,006	5,14E-06	9,20E-04	-0,31
TLN1	cg00833393	9	35 731 638	-0,063	2,83E-12	1,70E-10	1696643	0,007	2,13E-07	6,84E-05	-0,31
EPHX2	cg25399743	8	27 348 177	0,056	2,05E-05	9,30E-05	1709237	-0,013	4,60E-11	7,16E-08	-0,31
TMIGD2	cg20790056	19	4 303 012	0,076	1,10E-12	8,58E-11	2160577	-0,004	8,06E-07	2,06E-04	-0,31
NCAM1	cg12978214	11	113 145 174	-0,068	5,78E-09	8,05E-08	1676289	0,003	1,25E-04	1,09E-02	-0,31
ZNF518B	cg23995914	4	10 459 228	0,102	2,35E-16	6,27E-13	1682449	-0,007	1,77E-07	5,88E-05	-0,31
ITGAE	cg11011533	17	3 674 649	0,124	4,43E-13	4,53E-11	1683927	-0,004	1,08E-03	4,91E-02	-0,31
NCAM1	cg02323699	11	113 135 432	-0,075	7,26E-08	6,70E-07	1676289	0,003	1,25E-04	1,09E-02	-0,30
TGFBR2	cg13724812	3	30 689 835	0,106	1,02E-10	2,83E-09	2384241	-0,014	2,02E-06	4,37E-04	-0,30
GSTP1	cg25866895	11	67 350 326	-0,055	1,07E-05	5,19E-05	1679809	0,006	1,67E-04	1,34E-02	-0,30
ZNF518B	cg16295725	4	10 459 219	0,064	2,11E-12	1,37E-10	1682449	-0,007	1,77E-07	5,88E-05	-0,30
IGF1R	cg22375192	15	99 193 466	0,085	2,99E-10	6,85E-09	1744023	-0,008	1,21E-09	1,19E-06	-0,30

MATK	cg17880816	19	3 789 435	-0,051	2,41E-05	1,07E-04	2319000	0,009	2,00E-04	1,56E-02	-0,30
NT5E	cg17644557	6	86 159 103	0,098	1,04E-14	4,56E-12	1697220	-0,006	1,13E-06	2,73E-04	-0,30
SATB1	cg18808261	3	18 464 935	0,089	1,33E-06	8,17E-06	3247857	-0,007	7,00E-09	4,97E-06	-0,30
IFT140	cg26931990	16	1 661 230	0,096	2,13E-15	1,99E-12	1751559	-0,007	1,40E-07	5,07E-05	-0,30
SNX29	cg04635642	16	12 183 938	0,125	1,96E-09	3,27E-08	1670539	-0,007	4,15E-04	2,58E-02	-0,30
MAPK1	cg11335969	22	22 118 304	-0,059	9,33E-04	2,95E-03	2235283	0,006	5,38E-04	3,15E-02	-0,30
SLC7A6	cg03987842	16	68 321 858	0,063	6,68E-04	2,18E-03	1812559	-0,014	1,15E-08	7,16E-06	-0,30
IFT140	cg07013955	16	1 595 532	0,084	6,91E-07	4,60E-06	3241657	-0,007	2,82E-07	8,79E-05	-0,30
TMIGD2	cg22245273	19	4 302 448	0,054	3,42E-08	3,57E-07	2160577	-0,004	8,06E-07	2,06E-04	-0,30
ALDH5A1	cg25181693	6	24 495 846	0,058	1,22E-09	2,21E-08	2372398	-0,004	6,13E-06	1,06E-03	-0,30
ZNF629	cg22311615	16	30 798 776	0,075	1,23E-12	9,31E-11	1810891	-0,002	2,02E-04	1,57E-02	-0,30
PYGB	cg04348305	20	25 230 959	0,101	1,45E-13	2,14E-11	1778360	-0,006	8,56E-04	4,20E-02	-0,30
HOOK1	cg16378352	1	60 280 106	0,066	4,05E-08	4,10E-07	1741406	-0,004	4,11E-04	2,56E-02	-0,30
ACOT11	cg11837749	1	55 047 332	-0,056	5,79E-06	2,99E-05	1778373	0,007	5,68E-05	6,01E-03	-0,30
GAS7	cg18657751	17	9 834 056	-0,090	2,96E-07	2,21E-06	1745994	0,007	3,68E-04	2,38E-02	-0,30
RASSF4	cg02076355	10	45 474 372	-0,055	1,85E-07	1,49E-06	1690566	0,004	2,98E-05	3,81E-03	-0,30
GFI1	cg14179389	1	92 947 961	-0,055	5,06E-05	2,10E-04	1690420	0,006	2,39E-04	1,75E-02	-0,30
LTK	cg27021986	15	41 805 273	0,067	2,12E-11	7,94E-10	1679290	-0,005	4,55E-05	5,11E-03	-0,30
ROBO1	cg14467816	3	78 841 760	-0,077	3,55E-08	3,68E-07	1806790	-0,003	4,96E-07	1,40E-04	0,30
MCF2L	cg21331791	13	113 653 004	-0,056	2,00E-07	1,59E-06	3307483	-0,003	5,05E-05	5,52E-03	0,30
INF2	cg05018513	14	105 173 980	-0,073	2,25E-09	3,67E-08	1727248	-0,004	5,89E-05	6,19E-03	0,30
RHBDF2	cg20690125	17	74 476 978	0,108	1,12E-10	3,06E-09	1691717	0,006	6,81E-04	3,67E-02	0,30
RGS12	cg17569842	4	3 371 566	0,069	2,96E-08	3,17E-07	1722834	0,006	2,35E-06	4,96E-04	0,30
RB1	cg13431205	13	48 893 174	0,062	2,27E-06	1,30E-05	1786429	0,005	7,33E-04	3,84E-02	0,30
APBA2	cg22135269	15	29 338 250	-0,055	9,89E-06	4,83E-05	1723626	-0,010	4,50E-10	5,35E-07	0,30
IGFBP3	cg10094651	7	45 961 778	0,063	3,39E-09	5,16E-08	2396875	0,005	1,50E-07	5,36E-05	0,30
ESPN	cg10949430	1	6 496 243	-0,058	8,14E-09	1,07E-07	1765109	-0,013	8,36E-07	2,11E-04	0,30
IGFBP3	cg23455440	7	45 961 508	0,076	3,46E-11	1,17E-09	1746085	0,005	2,00E-05	2,80E-03	0,30
NBEAL2	cg02534363	3	47 050 950	0,098	5,90E-08	5,63E-07	1660629	0,010	3,96E-06	7,43E-04	0,30
SH3PXD2A	cg01428849	10	105 411 075	-0,071	1,00E-07	8,80E-07	1743103	-0,006	1,87E-05	2,64E-03	0,30
SH3PXD2A	cg08740008	10	105 373 505	-0,055	6,89E-07	4,59E-06	1743103	-0,006	1,87E-05	2,64E-03	0,30
PRRG2	cg04201347	19	50 086 849	-0,074	5,06E-07	3,51E-06	2064150	-0,004	7,99E-04	4,01E-02	0,30
LTBP3	cg03101763	11	65 319 844	-0,059	3,35E-11	1,14E-09	1805395	-0,012	6,47E-09	4,97E-06	0,30
LTBP4	cg22046408	19	41 106 164	0,098	1,14E-10	3,08E-09	1665219	0,006	7,70E-04	3,94E-02	0,30
MCF2L	cg22004422	13	113 740 730	-0,063	2,65E-07	2,02E-06	3307483	-0,003	5,05E-05	5,52E-03	0,30
CLEC11A	cg25309343	19	51 226 207	-0,066	9,75E-11	2,73E-09	1807359	-0,004	6,14E-04	3,42E-02	0,30
CRABP2	cg02363950	1	156 676 793	0,084	6,02E-11	1,84E-09	1690170	0,005	2,70E-04	1,90E-02	0,31
SLC7A8	cg21523574	14	23 595 597	-0,062	6,51E-07	4,37E-06	1807894	-0,003	6,95E-04	3,73E-02	0,31
ABR	cg05707655	17	908 690	0,096	1,61E-11	6,40E-10	1672878	0,006	5,60E-04	3,21E-02	0,31
SNX29	cg02946846	16	12 632 861	-0,070	2,99E-07	2,23E-06	1670539	-0,007	4,15E-04	2,58E-02	0,31
ELMO1	cg24237862	7	37 026 842	-0,059	8,98E-08	8,01E-07	3200299	-0,004	3,62E-04	2,36E-02	0,31
C1QTNF6	cg07573342	22	37 582 320	-0,077	7,52E-06	3,78E-05	1729288	-0,006	5,33E-04	3,12E-02	0,31
ADAP2	cg05052633	17	29 249 485	0,077	5,45E-12	2,78E-10	1763000	0,003	9,77E-04	4,60E-02	0,31

SDK2	cg26504421	17	71 339 361	-0,065	4,74E-06	2,51E-05	1676709	-0,007	6,79E-05	6,77E-03	0,31
INF2	cg04966159	14	105 173 801	-0,057	6,08E-08	5,77E-07	1727248	-0,004	5,89E-05	6,19E-03	0,31
MCF2L	cg12339531	13	113 650 822	-0,056	2,05E-07	1,62E-06	3307483	-0,003	5,05E-05	5,52E-03	0,31
CACHD1	cg17181043	1	64 961 535	-0,096	6,07E-08	5,77E-07	1738854	-0,005	1,55E-11	2,96E-08	0,32
ATP10A	cg01372572	15	25 941 836	-0,051	1,11E-06	6,98E-06	3307935	-0,004	2,14E-05	2,94E-03	0,32
MTHFD1L	cg21691367	6	151 325 642	-0,087	6,35E-08	5,99E-07	1772521	-0,002	4,79E-04	2,87E-02	0,32
ROBO1	cg15429180	3	79 137 306	-0,077	3,46E-07	2,53E-06	1806790	-0,003	4,96E-07	1,40E-04	0,32
CACHD1	cg25210796	1	65 089 808	-0,083	1,33E-06	8,15E-06	1738854	-0,005	1,55E-11	2,96E-08	0,32
SNX29	cg01223986	16	12 265 008	-0,067	4,18E-07	2,98E-06	1670539	-0,007	4,15E-04	2,58E-02	0,32
ENGASE	cg14609289	17	77 083 037	-0,051	3,23E-12	1,87E-10	1815366	-0,005	1,89E-04	1,48E-02	0,32
INF2	cg23956771	14	105 184 057	-0,079	1,94E-07	1,55E-06	1727248	-0,004	5,89E-05	6,19E-03	0,32
IGFBP3	cg10094651	7	45 961 778	0,063	3,39E-09	5,16E-08	1746085	0,005	2,00E-05	2,80E-03	0,32
CACHD1	cg11795488	1	65 038 492	-0,086	1,25E-07	1,06E-06	1738854	-0,005	1,55E-11	2,96E-08	0,32
SDK2	cg01691104	17	71 612 776	-0,066	2,32E-08	2,58E-07	1676709	-0,007	6,79E-05	6,77E-03	0,33
OSBPL5	cg23357130	11	3 181 928	0,071	3,21E-06	1,77E-05	1802151	0,008	8,45E-04	4,16E-02	0,33
SPIRE1	cg15920762	18	12 657 056	0,057	4,82E-07	3,37E-06	1757845	0,003	2,82E-04	1,97E-02	0,33
SATB2	cg10185119	2	200 325 426	0,064	8,20E-09	1,08E-07	1738657	0,002	2,09E-04	1,61E-02	0,33
LIMK2	cg07713946	22	31 675 144	0,101	1,24E-07	1,05E-06	2367671	0,001	5,87E-04	3,32E-02	0,33
CACHD1	cg24639703	1	64 986 155	-0,085	1,05E-05	5,08E-05	1738854	-0,005	1,55E-11	2,96E-08	0,33
CNN3	cg12613632	1	95 385 935	-0,073	3,38E-09	5,14E-08	1782439	-0,003	9,73E-08	3,68E-05	0,33
MCF2L	cg18289306	13	113 640 169	-0,070	2,72E-08	2,95E-07	3307483	-0,003	5,05E-05	5,52E-03	0,33
APEX1	cg16205058	14	20 924 369	-0,068	1,13E-10	3,07E-09	2319344	-0,006	5,31E-04	3,11E-02	0,33
CACHD1	cg21679730	1	65 047 485	-0,053	2,12E-07	1,67E-06	1738854	-0,005	1,55E-11	2,96E-08	0,33
FGR	cg03018771	1	27 940 619	0,074	1,45E-09	2,55E-08	2368318	0,017	8,84E-08	3,41E-05	0,33
LRRC16A	cg03372385	6	25 324 431	-0,065	7,85E-04	2,52E-03	3244461	-0,002	2,33E-04	1,72E-02	0,33
RB1	cg03085377	13	48 893 376	0,062	4,35E-06	2,32E-05	1786429	0,005	7,33E-04	3,84E-02	0,33
NDUFS2	cg17904988	1	161 168 451	-0,055	1,28E-06	7,87E-06	1789342	-0,003	5,05E-04	2,99E-02	0,33
PLEKHB1	cg02201052	11	73 357 741	-0,057	5,07E-09	7,23E-08	1783231	-0,006	7,93E-06	1,34E-03	0,33
IGFBP3	cg23455440	7	45 961 508	0,076	3,46E-11	1,17E-09	2396875	0,005	1,50E-07	5,36E-05	0,34
IFT140	cg01356115	16	1 614 807	-0,057	2,83E-07	2,13E-06	1751559	-0,007	1,40E-07	5,07E-05	0,34
INF2	cg00816970	14	105 181 035	-0,061	3,91E-08	3,99E-07	1727248	-0,004	5,89E-05	6,19E-03	0,34
IGFBP3	cg24772240	7	45 961 537	0,056	6,53E-09	8,92E-08	2396875	0,005	1,50E-07	5,36E-05	0,34
MCF2L	cg17113476	13	113 720 009	-0,059	3,91E-09	5,81E-08	3307483	-0,003	5,05E-05	5,52E-03	0,34
ROBO1	cg00906420	3	78 917 133	-0,103	9,33E-09	1,20E-07	1806790	-0,003	4,96E-07	1,40E-04	0,34
DCHS1	cg16770368	11	6 673 784	-0,075	3,43E-10	7,68E-09	1739640	-0,003	2,57E-08	1,38E-05	0,34
CACHD1	cg00825690	1	64 960 202	-0,081	2,34E-07	1,82E-06	1738854	-0,005	1,55E-11	2,96E-08	0,34
SATB2	cg17204275	2	200 321 136	0,056	3,49E-12	1,99E-10	1738657	0,002	2,09E-04	1,61E-02	0,34
ABR	cg01841306	17	908 923	0,081	5,39E-07	3,71E-06	1672878	0,006	5,60E-04	3,21E-02	0,34
IGFBP3	cg09619271	7	45 961 289	0,087	2,87E-10	6,62E-09	2396875	0,005	1,50E-07	5,36E-05	0,34
LILRB1	cg02340056	19	55 141 979	0,068	3,34E-08	3,51E-07	1708248	0,002	2,52E-04	1,80E-02	0,34
P2RX6	cg01653494	22	21 372 286	-0,065	9,68E-10	1,82E-08	3243924	-0,003	6,80E-05	6,77E-03	0,34
CNN3	cg25311623	1	95 375 671	-0,106	2,69E-07	2,04E-06	1782439	-0,003	9,73E-08	3,68E-05	0,34
ITGB2	cg18012089	21	46 327 720	0,060	2,97E-05	1,30E-04	2175912	0,006	1,25E-04	1,09E-02	0,34

GPC2	cg01942654	7	99 773 786	-0,053	1,23E-06	7,64E-06	1651642	-0,003	8,65E-04	4,23E-02	0,35
RB1	cg07880715	13	48 895 478	0,052	1,52E-07	1,25E-06	1786429	0,005	7,33E-04	3,84E-02	0,35
BLK	cg24861686	8	11 418 058	-0,051	8,19E-11	2,36E-09	1668277	-0,006	5,14E-06	9,20E-04	0,35
SNX29	cg04637129	16	12 212 548	-0,066	1,32E-07	1,11E-06	1670539	-0,007	4,15E-04	2,58E-02	0,35
MCF2L	cg20074340	13	113 651 420	-0,053	3,06E-08	3,25E-07	3307483	-0,003	5,05E-05	5,52E-03	0,35
SDK2	cg22121495	17	71 420 061	-0,051	6,47E-06	3,31E-05	1676709	-0,007	6,79E-05	6,77E-03	0,35
CACHD1	cg10381153	1	65 062 855	-0,088	2,99E-08	3,20E-07	1738854	-0,005	1,55E-11	2,96E-08	0,35
INF2	cg05210373	14	105 173 786	-0,063	1,66E-06	9,90E-06	1727248	-0,004	5,89E-05	6,19E-03	0,35
IGF1R	cg16735875	15	99 367 484	-0,053	2,55E-08	2,80E-07	1744023	-0,008	1,21E-09	1,19E-06	0,35
LRRC16A	cg23737208	6	25 499 464	-0,052	3,20E-04	1,12E-03	3244461	-0,002	2,33E-04	1,72E-02	0,35
GIPC3	cg25597797	19	3 589 762	-0,116	3,23E-07	2,39E-06	3253728	-0,002	8,79E-06	1,46E-03	0,35
SDK2	cg20458433	17	71 463 069	-0,065	1,56E-07	1,28E-06	1676709	-0,007	6,79E-05	6,77E-03	0,35
IGFBP3	cg24772240	7	45 961 537	0,056	6,53E-09	8,92E-08	1746085	0,005	2,00E-05	2,80E-03	0,35
RPL13	cg02732508	16	89 628 853	-0,071	2,03E-05	9,20E-05	2413278	-0,006	8,54E-06	1,43E-03	0,35
LRRC16A	cg21272716	6	25 619 939	-0,060	4,86E-06	2,56E-05	3244461	-0,002	2,33E-04	1,72E-02	0,36
SERPINE2	cg20793341	2	224 897 527	-0,073	3,83E-08	3,92E-07	1655595	-0,007	1,25E-08	7,58E-06	0,36
SDK2	cg24527434	17	71 544 677	-0,063	3,13E-06	1,73E-05	1676709	-0,007	6,79E-05	6,77E-03	0,36
IGFBP3	cg26434048	7	45 961 569	0,064	8,33E-08	7,52E-07	1746085	0,005	2,00E-05	2,80E-03	0,36
IGFBP3	cg02120774	7	45 961 473	0,058	9,57E-11	2,68E-09	1746085	0,005	2,00E-05	2,80E-03	0,36
TM6SF1	cg09682213	15	83 777 082	0,057	6,54E-06	3,34E-05	1784256	0,005	7,34E-05	7,16E-03	0,36
IFT140	cg03515656	16	1 600 304	-0,086	1,86E-07	1,49E-06	3241657	-0,007	2,82E-07	8,79E-05	0,36
ABLIM1	cg03850986	10	116 408 382	-0,097	1,14E-12	8,76E-11	2396672	-0,008	9,88E-06	1,61E-03	0,36
SATB2	cg03163783	2	200 326 591	0,090	4,09E-10	8,89E-09	1738657	0,002	2,09E-04	1,61E-02	0,36
PDE9A	cg21688288	21	44 181 258	-0,050	7,99E-07	5,22E-06	1683063	-0,004	3,02E-06	6,05E-04	0,36
IGFBP3	cg26434048	7	45 961 569	0,064	8,33E-08	7,52E-07	2396875	0,005	1,50E-07	5,36E-05	0,36
SPEG	cg24685587	2	220 300 568	-0,061	1,32E-05	6,27E-05	1740052	-0,003	6,40E-05	6,51E-03	0,37
ROBO1	cg08147563	3	79 028 263	-0,075	1,97E-07	1,56E-06	1806790	-0,003	4,96E-07	1,40E-04	0,37
MCF2L	cg09508934	13	113 717 379	-0,060	4,56E-08	4,53E-07	3307483	-0,003	5,05E-05	5,52E-03	0,37
PLAG1	cg21448513	8	57 103 638	-0,077	2,21E-08	2,48E-07	2287653	-0,002	3,27E-05	4,09E-03	0,37
NLGN2	cg16950726	17	7 317 514	-0,064	1,04E-06	6,56E-06	1764158	-0,004	9,47E-06	1,55E-03	0,37
CYB561	cg22122808	17	61 511 683	0,069	8,56E-06	4,25E-05	1771179	0,005	2,74E-04	1,92E-02	0,37
IGF1R	cg05110803	15	99 385 323	-0,094	3,45E-10	7,71E-09	1744023	-0,008	1,21E-09	1,19E-06	0,37
CLCF1	cg11169848	11	67 142 030	0,062	2,00E-07	1,58E-06	1661197	0,005	7,40E-04	3,85E-02	0,37
UBASH3B	cg25994988	11	122 652 382	-0,079	6,53E-14	1,30E-11	1653856	-0,005	6,27E-07	1,65E-04	0,38
CYB561	cg18499750	17	61 511 735	0,074	3,13E-14	8,30E-12	1771179	0,005	2,74E-04	1,92E-02	0,38
PPFIBP2	cg26427534	11	7 659 067	-0,087	2,80E-07	2,11E-06	1675656	-0,008	7,09E-04	3,77E-02	0,38
FGR	cg03018771	1	27 940 619	0,074	1,45E-09	2,55E-08	1795158	0,015	2,30E-07	7,32E-05	0,38
APEX1	cg16205058	14	20 924 369	-0,068	1,13E-10	3,07E-09	1661886	-0,008	5,92E-04	3,34E-02	0,38
IGFBP3	cg09619271	7	45 961 289	0,087	2,87E-10	6,62E-09	1746085	0,005	2,00E-05	2,80E-03	0,38
ROBO1	cg01375651	3	79 026 069	-0,061	1,61E-08	1,90E-07	1806790	-0,003	4,96E-07	1,40E-04	0,38
TNNT3	cg09833701	11	1 959 051	-0,082	4,13E-09	6,09E-08	2334080	-0,003	2,91E-07	8,93E-05	0,38
SDK2	cg19786808	17	71 431 877	-0,055	8,43E-07	5,47E-06	1676709	-0,007	6,79E-05	6,77E-03	0,38
KLHL29	cg15736743	2	23 768 363	-0,058	7,22E-06	3,65E-05	3178406	-0,004	2,58E-05	3,36E-03	0,38

PDE9A	cg00118229	21	44 088 420	-0,073	1,21E-07	1,04E-06	1683063	-0,004	3,02E-06	6,05E-04	0,39
SERPINF1	cg06940127	17	1 665 303	-0,067	2,66E-10	6,20E-09	2141482	-0,002	5,41E-04	3,15E-02	0,39
SYTL3	cg12934258	6	159 084 599	0,073	8,86E-09	1,15E-07	1720623	0,009	2,69E-06	5,47E-04	0,39
PRSS23	cg23771366	11	86 510 998	0,051	8,45E-06	4,20E-05	1797776	0,010	1,56E-04	1,29E-02	0,39
ROBO1	cg24512093	3	78 698 151	-0,117	2,23E-09	3,65E-08	1806790	-0,003	4,96E-07	1,40E-04	0,39
PTK7	cg08558323	6	43 097 370	-0,055	8,16E-11	2,36E-09	2353202	-0,002	8,48E-08	3,30E-05	0,39
SDK2	cg03695433	17	71 332 907	-0,074	1,60E-07	1,31E-06	1676709	-0,007	6,79E-05	6,77E-03	0,40
ITGAE	cg06468347	17	3 705 875	-0,057	2,79E-04	9,85E-04	1683927	-0,004	1,08E-03	4,91E-02	0,40
SDK2	cg14295306	17	71 355 182	-0,087	1,21E-08	1,50E-07	1676709	-0,007	6,79E-05	6,77E-03	0,40
MMP28	cg11992940	17	34 094 454	-0,089	2,44E-10	5,79E-09	2399016	-0,007	1,02E-12	3,74E-09	0,40
IFT140	cg03515656	16	1 600 304	-0,086	1,86E-07	1,49E-06	1751559	-0,007	1,40E-07	5,07E-05	0,41
CACNA2D2	cg26325335	3	50 402 333	0,101	2,53E-11	9,13E-10	1711049	0,006	2,31E-05	3,10E-03	0,41
TNNT3	cg06124793	11	1 939 725	-0,053	5,09E-11	1,60E-09	2334080	-0,003	2,91E-07	8,93E-05	0,41
ROBO1	cg15442678	3	79 065 762	-0,091	4,84E-10	1,02E-08	1806790	-0,003	4,96E-07	1,40E-04	0,41
MCF2L	cg21942572	13	113 707 517	-0,055	2,87E-10	6,61E-09	3307483	-0,003	5,05E-05	5,52E-03	0,41
BTBD11	cg11362622	12	107 836 508	-0,085	6,31E-08	5,96E-07	1705066	-0,014	9,52E-09	6,22E-06	0,41
LDLRAP1	cg11214576	1	25 869 068	-0,059	6,54E-07	4,39E-06	1809040	-0,008	6,75E-07	1,74E-04	0,41
TNNT3	cg04052013	11	1 959 246	-0,054	2,62E-09	4,16E-08	2334080	-0,003	2,91E-07	8,93E-05	0,41
SDK2	cg20614854	17	71 331 689	-0,081	2,92E-07	2,19E-06	1676709	-0,007	6,79E-05	6,77E-03	0,41
PGAP2	cg01134526	11	3 846 965	-0,061	4,01E-07	2,88E-06	1755405	-0,005	8,57E-05	8,07E-03	0,42
IGF1R	cg02613818	15	99 236 767	-0,058	1,06E-06	6,70E-06	1744023	-0,008	1,21E-09	1,19E-06	0,42
ABR	cg06237543	17	1 083 824	0,051	2,93E-05	1,28E-04	1672878	0,006	5,60E-04	3,21E-02	0,42
ENTPD3	cg12433782	3	40 427 210	-0,060	3,98E-07	2,85E-06	1775114	-0,002	3,76E-04	2,42E-02	0,42
IGF1R	cg18542304	15	99 288 216	-0,057	3,81E-08	3,91E-07	1744023	-0,008	1,21E-09	1,19E-06	0,42
IGF1R	cg01284192	15	99 500 473	-0,066	2,43E-08	2,68E-07	1744023	-0,008	1,21E-09	1,19E-06	0,42
TNNT3	cg08089153	11	1 956 292	-0,090	5,70E-09	7,96E-08	2334080	-0,003	2,91E-07	8,93E-05	0,42
IGF1R	cg11145296	15	99 400 281	-0,069	1,07E-08	1,35E-07	1744023	-0,008	1,21E-09	1,19E-06	0,42
KCNT1	cg13481132	9	138 606 811	0,086	1,13E-13	1,83E-11	1702255	0,004	5,85E-04	3,32E-02	0,42
AEBP1	cg08495088	7	44 143 290	-0,060	1,98E-07	1,57E-06	1736178	-0,004	5,23E-05	5,71E-03	0,42
IGFBP3	cg02120774	7	45 961 473	0,058	9,57E-11	2,68E-09	2396875	0,005	1,50E-07	5,36E-05	0,42
NRCAM	cg16073236	7	107 789 943	-0,065	5,08E-07	3,52E-06	1722809	-0,008	3,07E-16	3,11E-12	0,42
BTBD11	cg24671734	12	107 909 364	-0,070	2,10E-08	2,37E-07	1705066	-0,014	9,52E-09	6,22E-06	0,42
GPR56	cg09109520	16	57 673 258	0,070	1,27E-10	3,38E-09	2384122	0,018	5,05E-06	9,13E-04	0,42
BTBD11	cg04076774	12	107 954 581	-0,061	4,81E-08	4,74E-07	1705066	-0,014	9,52E-09	6,22E-06	0,43
MYO15B	cg09913882	17	73 612 981	-0,058	1,93E-07	1,54E-06	3248649	-0,009	2,39E-05	3,18E-03	0,43
IGF1R	cg21572688	15	99 391 577	-0,050	1,20E-07	1,02E-06	1744023	-0,008	1,21E-09	1,19E-06	0,43
CDH23	cg04567334	10	73 408 185	-0,072	6,44E-08	6,06E-07	1783149	-0,010	4,44E-08	2,04E-05	0,43
IGF1R	cg05169988	15	99 204 912	-0,095	1,81E-09	3,07E-08	1744023	-0,008	1,21E-09	1,19E-06	0,43
BACH2	cg17066529	6	90 754 140	-0,066	1,44E-05	6,76E-05	1670695	-0,007	2,54E-10	3,31E-07	0,43
CR2	cg26858300	1	207 643 142	-0,063	1,54E-09	2,68E-08	2369666	-0,004	6,46E-08	2,78E-05	0,43
SPEG	cg20456243	2	220 352 476	-0,108	6,71E-08	6,28E-07	1740052	-0,003	6,40E-05	6,51E-03	0,43
PCSK5	cg13438337	9	78 774 369	-0,067	6,15E-07	4,16E-06	1767934	-0,012	1,52E-13	7,67E-10	0,43
CDH23	cg08734090	10	73 479 514	-0,055	2,58E-07	1,97E-06	1783149	-0,010	4,44E-08	2,04E-05	0,43

BTBD11	cg19939178	12	107 976 961	-0,073	3,95E-07	2,84E-06	1705066	-0,014	9,52E-09	6,22E-06	0,44
SCRN1	cg22799757	7	29 997 642	-0,127	3,52E-08	3,66E-07	1756439	-0,008	3,11E-07	9,34E-05	0,44
APBA2	cg07699300	15	29 392 964	-0,067	4,20E-06	2,25E-05	1723626	-0,010	4,50E-10	5,35E-07	0,44
SDK2	cg19787125	17	71 532 174	-0,074	8,44E-06	4,19E-05	1676709	-0,007	6,79E-05	6,77E-03	0,44
SERINC5	cg17542330	5	79 445 875	-0,060	3,30E-07	2,43E-06	1789464	-0,008	1,20E-07	4,38E-05	0,45
CDH23	cg15077792	10	73 324 481	-0,062	1,31E-10	3,46E-09	1783149	-0,010	4,44E-08	2,04E-05	0,45
SCRN1	cg18395623	7	29 986 771	-0,086	5,23E-12	2,69E-10	1756439	-0,008	3,11E-07	9,34E-05	0,45
LRRC16A	cg09110394	6	25 301 880	-0,095	3,00E-09	4,66E-08	3244461	-0,002	2,33E-04	1,72E-02	0,45
APBA2	cg08121164	15	29 404 922	-0,050	5,39E-06	2,81E-05	1723626	-0,010	4,50E-10	5,35E-07	0,45
NPM3	cg05901193	10	103 541 206	-0,053	5,41E-07	3,72E-06	2110252	-0,009	1,58E-09	1,46E-06	0,45
IGF1R	cg20388729	15	99 200 492	-0,092	1,29E-06	7,94E-06	1744023	-0,008	1,21E-09	1,19E-06	0,45
CDH23	cg04161859	10	73 404 657	-0,076	1,19E-07	1,02E-06	1783149	-0,010	4,44E-08	2,04E-05	0,46
BTBD11	cg10673246	12	108 003 445	-0,051	1,03E-07	9,00E-07	1705066	-0,014	9,52E-09	6,22E-06	0,46
FLNB	cg20840069	3	58 147 905	-0,062	8,30E-07	5,40E-06	1664922	-0,013	3,39E-12	9,15E-09	0,46
ADD2	cg02674324	2	70 932 722	-0,071	3,01E-08	3,22E-07	1833858	-0,008	9,29E-10	9,90E-07	0,46
ADD2	cg06915226	2	70 960 959	-0,070	1,09E-06	6,85E-06	1833858	-0,008	9,29E-10	9,90E-07	0,46
FBLN2	cg15097422	3	13 597 750	-0,075	8,39E-10	1,62E-08	2390919	-0,009	1,62E-17	2,18E-13	0,47
SOX13	cg07142667	1	204 041 849	0,054	1,50E-07	1,24E-06	1683755	0,006	3,30E-05	4,11E-03	0,47
NRCAM	cg23543795	7	107 965 081	-0,096	3,11E-08	3,30E-07	1722809	-0,008	3,07E-16	3,11E-12	0,47
FBLN2	cg15097422	3	13 597 750	-0,075	8,39E-10	1,62E-08	1774602	-0,011	2,16E-19	4,36E-15	0,47
CDH23	cg05848785	10	73 480 942	-0,059	1,81E-08	2,10E-07	1783149	-0,010	4,44E-08	2,04E-05	0,48
BTBD11	cg27431274	12	107 979 028	-0,100	9,60E-10	1,81E-08	1705066	-0,014	9,52E-09	6,22E-06	0,48
TBC1D4	cg24425149	13	75 991 251	-0,088	9,00E-13	7,42E-11	1688098	-0,010	1,95E-07	6,35E-05	0,48
PDE9A	cg21688288	21	44 181 258	-0,050	7,99E-07	5,22E-06	2306540	-0,015	1,61E-11	2,96E-08	0,48
ADD2	cg24938166	2	70 941 682	-0,064	2,21E-08	2,47E-07	1833858	-0,008	9,29E-10	9,90E-07	0,48
TARBP1	cg02838825	1	234 613 490	-0,057	1,70E-06	1,01E-05	1803036	-0,008	3,47E-06	6,74E-04	0,49
CDH23	cg09501025	10	73 312 955	-0,098	1,74E-07	1,41E-06	1783149	-0,010	4,44E-08	2,04E-05	0,49
BTBD11	cg00174508	12	107 774 298	-0,063	7,01E-10	1,39E-08	1705066	-0,014	9,52E-09	6,22E-06	0,49
CDH23	cg03033796	10	73 445 156	-0,077	7,09E-09	9,56E-08	1783149	-0,010	4,44E-08	2,04E-05	0,50
NRCAM	cg24429533	7	107 886 896	-0,083	2,57E-08	2,81E-07	1722809	-0,008	3,07E-16	3,11E-12	0,50
APBA2	cg07148167	15	29 403 393	-0,052	2,01E-07	1,59E-06	1723626	-0,010	4,50E-10	5,35E-07	0,50
CHD7	cg21340143	8	61 676 203	-0,062	1,21E-07	1,03E-06	1677376	-0,006	8,96E-05	8,34E-03	0,50
FBLN2	cg16157615	3	13 607 231	-0,078	1,59E-10	4,06E-09	1774602	-0,011	2,16E-19	4,36E-15	0,50
NRCAM	cg25994871	7	107 967 614	-0,080	2,90E-09	4,52E-08	1722809	-0,008	3,07E-16	3,11E-12	0,50
PCSK5	cg14178077	9	78 777 801	-0,106	8,02E-08	7,29E-07	1767934	-0,012	1,52E-13	7,67E-10	0,51
KBTBD11	cg20849109	8	1 954 777	-0,085	2,35E-09	3,81E-08	1784630	-0,006	7,26E-04	3,83E-02	0,51
PCSK5	cg21172497	9	78 510 119	-0,091	6,55E-07	4,40E-06	1767934	-0,012	1,52E-13	7,67E-10	0,51
CDH23	cg22646408	10	73 256 318	-0,094	3,99E-09	5,91E-08	1783149	-0,010	4,44E-08	2,04E-05	0,51
FBLN2	cg16157615	3	13 607 231	-0,078	1,59E-10	4,06E-09	2390919	-0,009	1,62E-17	2,18E-13	0,51
ZNF256	cg00541956	19	58 455 018	-0,054	1,39E-09	2,47E-08	1757408	-0,009	5,63E-07	1,54E-04	0,51
CDH23	cg13714026	10	73 385 503	-0,065	6,73E-08	6,29E-07	1783149	-0,010	4,44E-08	2,04E-05	0,52
ACTN1	cg15364504	14	69 416 134	-0,053	4,01E-08	4,07E-07	2232177	-0,017	1,94E-07	6,35E-05	0,52
PDE9A	cg00118229	21	44 088 420	-0,073	1,21E-07	1,04E-06	2306540	-0,015	1,61E-11	2,96E-08	0,52

NRCAM	cg16073236	7	107 789 943	-0,065	5,08E-07	3,52E-06	2411236	-0,020	2,37E-21	9,60E-17	0,53
ABLIM1	cg27290215	10	116 392 933	-0,083	6,33E-10	1,28E-08	1785424	-0,018	9,63E-12	1,95E-08	0,53
FBLN2	cg14789121	3	13 675 521	-0,053	5,79E-10	1,18E-08	2390919	-0,009	1,62E-17	2,18E-13	0,53
NRCAM	cg25311470	7	107 950 866	-0,064	5,95E-11	1,82E-09	1722809	-0,008	3,07E-16	3,11E-12	0,54
DLL1	cg13250324	6	170 591 863	-0,066	4,71E-07	3,30E-06	1743373	-0,006	1,96E-06	4,28E-04	0,55
PCSK5	cg14349078	9	78 547 359	-0,123	4,23E-09	6,20E-08	1767934	-0,012	1,52E-13	7,67E-10	0,56
FBLN2	cg14789121	3	13 675 521	-0,053	5,79E-10	1,18E-08	1774602	-0,011	2,16E-19	4,36E-15	0,57
NRCAM	cg23543795	7	107 965 081	-0,096	3,11E-08	3,30E-07	2411236	-0,020	2,37E-21	9,60E-17	0,58
ABLIM1	cg00950497	10	116 393 423	-0,050	3,32E-08	3,49E-07	1785424	-0,018	9,63E-12	1,95E-08	0,59
FLNB	cg01361263	3	58 051 763	-0,071	2,83E-07	2,13E-06	1664922	-0,013	3,39E-12	9,15E-09	0,59
NRCAM	cg25994871	7	107 967 614	-0,080	2,90E-09	4,52E-08	2411236	-0,020	2,37E-21	9,60E-17	0,59
NRCAM	cg00280235	7	107 797 076	-0,112	2,02E-12	1,33E-10	1722809	-0,008	3,07E-16	3,11E-12	0,59
NRCAM	cg25311470	7	107 950 866	-0,064	5,95E-11	1,82E-09	2411236	-0,020	2,37E-21	9,60E-17	0,60
NRCAM	cg24429533	7	107 886 896	-0,083	2,57E-08	2,81E-07	2411236	-0,020	2,37E-21	9,60E-17	0,61
NRCAM	cg00280235	7	107 797 076	-0,112	2,02E-12	1,33E-10	2411236	-0,020	2,37E-21	9,60E-17	0,64
ACTN1	cg20505457	14	69 388 953	-0,055	1,40E-06	8,54E-06	2232177	-0,017	1,94E-07	6,35E-05	0,68
ABLIM1	cg03850986	10	116 408 382	-0,097	1,14E-12	8,76E-11	1785424	-0,018	9,63E-12	1,95E-08	0,69

Supplementary Table 3. List of enriched functional terms sorted by p-value. The results were generated with g:Profiler and are graphically depicted in Fig. 4C.

Query	query	p.value	term.size	query.size	overlap.siz	precision	recall	term.id	subgr	term.name
CD8	hypomethylation	0,00023	155	82	8	0,098	0,052	GO:0002263	22	cell activation involved in immune response
CD8	hypomethylation	0,00031	224	82	9	0,11	0,04	GO:0018108	26	peptidyl-tyrosine phosphorylation
CD8	hypomethylation	0,00108	854	92	16	0,174	0,019	GO:0051336	4	regulation of hydrolase activity
CD8	hypermethylation	0,00242	82	103	5	0,049	0,061	KEGG:04520	4	Adherens junction
CD8	hypomethylation	0,00449	221	105	8	0,076	0,036	KEGG:04810	13	Regulation of actin cytoskeleton
CD8	hypomethylation	0,0106	90	97	5	0,052	0,056	KEGG:05215	5	Prostate cancer
CD8	hypomethylation	0,0134	696	105	14	0,133	0,02	GO:0042060	28	wound healing
CD8	hypermethylation	0,0153	536	138	14	0,101	0,026	GO:0010942	5	positive regulation of cell death
CD8	hypermethylation	0,0215	270	142	10	0,07	0,037	GO:0030098	8	lymphocyte differentiation
CD8	hypomethylation	0,0222	122	91	6	0,066	0,049	GO:0008360	10	regulation of cell shape
CD8	hypomethylation	0,0273	597	97	12	0,124	0,02	GO:0031347	12	regulation of defense response
CD8	hypermethylation	0,0279	784	142	17	0,12	0,022	GO:0007417	2	central nervous system development
CD8	hypomethylation	0,0347	562	105	12	0,114	0,021	GO:0007596	29	blood coagulation
CD8	hypomethylation	0,0347	562	105	12	0,114	0,021	GO:0030029	14	actin filament-based process
CD8	hypomethylation	0,0349	599	84	11	0,131	0,018	GO:0001816	9	cytokine production
CD8	hypermethylation	0,0442	486	145	13	0,09	0,027	GO:0030036	9	actin cytoskeleton organization

Supplementary Table 4. oPOSSUM analysis of transcription factor binding sites in gene promoters with inverse correlation of methylation and expression (log value >6).

TF	Family	Target gene hits	Target gene non-hits	Background gene hits	Background gene non-hits	Target TFBS hits	Background TFBS hits	Z-score*	Fisher score**
**Klf4**	BetaBetaAlpha-zinc finger	430	190	13645	11107	3375	88731	20.068	28.227
**SP1**	BetaBetaAlpha-zinc finger	383	237	12391	12361	3033	79797	18.872	19.214
**MZF1(1-4)**	BetaBetaAlpha-zinc finger	494	126	16863	7889	7431	201000	17.058	22.637
**MZF1(5-13)**	BetaBetaAlpha-zinc finger	396	224	13425	11327	2870	77649	13.560	13.823
**SPIB**	Ets	507	113	17547	7205	6336	175639	11.758	21.472
**ZNF354C**	BetaBetaAlpha-zinc finger	501	119	17566	7186	7486	207738	11.607	17.787
**RUNX1**	Runt	392	228	13641	11111	2157	58648	11.536	10.368
**ELF5**	Ets	475	145	16547	8205	4249	117435	11.482	16.168
**Myb**	Myb	399	221	13518	11234	2168	58600	10.621	14.123
**FEV**	Ets	454	166	15402	9350	2978	81722	10.121	18.785

* The Z-score is a normalized difference between the actual rate of occurrence of a TFBS in the target gene set and the expected rate of occurrence (computed from a set of background genes).

**The Fisher score is based on one-tailed Fisher exact test.

Supplementary Table 5. Oligonucleotides used for the Sequenom MassArray and RT-PCR experiments.

Amplicon	Sense	Antisense	Assay
IFNG	aggaagagagTTTAAAATGATTGTTTATAAGAGATGATAG	cagtaatacgactcactatagggagaaggctCAACAACCAAAAAAAATACAACACA	MassARRAY
GZMH	aggaagagagATTTGTTGGGTGTGTGTAATTTTGT	cagtaatacgactcactatagggagaaggctAAACCAAAAATATCTCCTTTTCTCAA	MassARRAY
CCR7	aggaagagagGTTATTGGTGATGTTGAAGTTGGTT	cagtaatacgactcactatagggagaaggctTCCATCCCAAAACTCCTATACAATA	MassARRAY
CD248	aggaagagagGGGGTGTAGATTTTTTTTATGGAGT	cagtaatacgactcactatagggagaaggctTTTTACTAAATCCTCCCAAAACCTC	MassARRAY
SATB1	aggaagagagGGGATTATATTTTTTTTGATTGGAG	cagtaatacgactcactatagggagaaggctCTAAAAAATTCCCAAAACTTCCACT	MassARRAY
CD27	aggaagagagTATAAAAGTGAGGAGTGAGTAAGGGTT	cagtaatacgactcactatagggagaaggctACCACAAAACACCCTTAAAAAATTA	MassARRAY
IFNG	TGGAAAGAGGAGAGTGACAG	TGGTCTCCACACTCTTTTGG	RT-qPCR
GZMH	TGGCGGCATCCTAGTGAGAA	GCCCCCAAGGTGACATTTATG	RT-qPCR
CCR7	CTTGTCATTTTCCAGGTATGCC	GGAGCACAAAGACTCGAACA	RT-qPCR
CD248	GCTGGACAGATGGCTTCC	GTGTATCCTTGGTCACGAGC	RT-qPCR
SATB1	CCATGTTCCAGCAGAGCAGATT	TCTGTGGCTGCTGCTGTG	RT-qPCR
CD27	GGCCACCCCAAAGATCCC	TCGTTGATGGAGGAACAGGG	RT-qPCR
